# Isolation and Genome Analysis of *Serratia ureilytica* T6, a Heavy Metal(loid)-Resistant and Plant Growth-Promoting Bacterium, from Rice Soil

**DOI:** 10.3390/microorganisms13122857

**Published:** 2025-12-16

**Authors:** Syed Muhammad Azam, Ziting Lin, Yanqing Bai, Yijia Fu, Hend Alwathnani, Guo-Hong Liu, Christopher Rensing

**Affiliations:** 1 Institute of Environmental Microbiology, College of Resources and Environment, Fujian Agriculture and Forestry University, Fuzhou 350002, China; syedazamfafu@gmail.com (S.M.A.);; 2 Department of Botany and Microbiology, King Saud University, Riyadh 11451, Saudi Arabia; 3 Institute of Resources, Environment and Soil Fertilizer, Fujian Academy of Agricultural Sciences, Fuzhou 350003, China

**Keywords:** *Serratia ureilytica* T6, lead and zinc resistance, PGPB, genome-wide analysis, POD, SOD, CAT

## Abstract

Lead and zinc pollution is a prevalent issue in agricultural soils surrounding lead and zinc mines, posing a serious risk to crop growth and soil health. Heavy metal-resistant, plant growth-promoting bacteria (PGPB) capable of supporting plant development under high metal exposure have significant potential for mitigating these deleterious effects. Here we isolated and identified the Pb- and Zn-resistant and plant growth-promoting bacterial strain *Serratia ureilytica* T6 based on 16S rRNA and average nucleotide identity (ANI) analysis. Furthermore, 14 strains (T1–T14) from a rice paddy soil irrigated by Pb-Zn mine effluent were isolated and identified, and their phytopromoting characteristics were determined. Genome analysis of *S. ureilytica* T6 showed a genome size of 5,102,941 bp, with G + C content of 59.74%. A total of 4822 genes were annotated by RAST, among which 15 genes were putatively associated with Pb-Zn resistance. The genome of *S. ureilytica* T6 was found to possess multiple genes associated with probiotic properties by a comparative analysis of KEGG, GO, and COG databases. Several taxonomic identifications of *S. ureilytica* T6 revealed that strain T6 is Gram-negative, facultative anaerobic and motile. The pH growth range of *S. ureilytica* T6 was between 4.00 and 9.50; temperature growth range was 4–37 °C; NaCl tolerance was 0–9%. *S. ureilytica* T6 displayed a high tolerance to a variety of heavy metals, with minimum inhibitory concentrations of 1.5 and 9 mmol·L^−1^ for Pb and Zn. *S. ureilytica* T6 can utilize a variety of carbon sources and nitrogen sources. T6 has the ability to produce indole-3-acetic acid (IAA), siderophore, and phosphorus and potassium solubilization, and it was initially judged that strain T6 has the potential for plant growth-promoting ability. Different plant growth-promoting effects of T6 inoculations were observed in improving rice biomass, plant height, etc. We observed that with increasing Pb and Zn stress, SOD activity first increased and then decreased, while POD and CAT activities gradually decreased. The addition of *S. ureilytica* T6 significantly enhanced the activities of SOD, POD, and CAT in rice seedlings under low to moderate Pb and Zn stress but had no significant effect under high concentrations (150 mg·L^−1^) of Pb or Zn. In addition, *S. ureilytica* T6 has the potential to be used as a phytoremediation tool.

## 1. Introduction

Lead (Pb) and zinc (Zn) contamination in soils has become a serious environmental problem in China and worldwide due to rapid industrialization, mining activities, and extensive use of these metals in various industries. Large areas of agricultural land have been affected by heavy metal accumulation, leading to soil degradation and potential entry of these toxic metals into the food chain. The widespread presence of Pb and Zn in soils threatens not only crop safety and productivity but also human health through the consumption of contaminated agricultural products. The persistence and bioaccumulation of these metals in soil ecosystems highlight the urgent need for effective remediation strategies to mitigate their adverse effects. China is vast and rich in heavy metal mineral resources, among which lead, cadmium, and zinc are particularly abundant. The reserves of lead and zinc rank second in the world, only behind Australia [1]. According to data released by the National Bureau of Statistics, in 2016, China’s lead ore reserves amounted to 18.086 million tons (metal content), and zinc ore reserves reached 44.391 million tons (metal content) [2]. In a typical lead mining area around Huize, Yunnan, the Pb and Zn contents in agricultural soils were 512 and 1761 mg·kg^−1,^ respectively, which is more than ten times higher than the provincial soil background values of 40.60 and 89.7 mg·kg^−1^ [3]. A study on farmland soils around lead–zinc mines in Yulan, Yongchang, and Shanshulin in northwest Guizhou found Pb and Zn contents of 960.38 and 2122.27 mg·kg^−1^, which exceeded the background values of Guizhou and Yunnan by more than 20 times, significantly surpassing the soil pollution risk control standards for high agricultural land [4]. Increased Zn and Pb concentrations can inhibit plant root growth, affect antioxidant responses, and induce oxidative and membrane damage, resulting in diminishing plant growth and ecosystem stability [5,6].

In the representative E-22 rice cultivar, lead toxicity was observed to reduce root length (17%), shoot length (16%), biological yield (55%), and levels of chlorophyll a and b (30%). Furthermore, lead toxicity affected reactive oxygen species (ROS) levels, such as malondialdehyde and H_2_O_2_, while lowering the activities of antioxidant enzymes [4,7]. Heavy metals include essential metals (Co, Cu, Mn, Zn, Fe, and Cr), non-essential metals (Ba, Al, Li, and Zr), less toxic metals (Sn), and highly toxic metals (As, Hg, Pb, and Cd) [8]. Zn and Pb, two contaminants in soils, disrupt microbial community structure and enzymatic activities, reducing nutrient-cycling efficacy and impairing overall soil ecological function [9,10]. Bacteria defy toxic metals mainly by means of efflux pumps, which actively export metal ions from the cytoplasm (e.g., RND systems, P-type ATPases, CDF transporters), and secondly through metal sequestration, where intracellular proteins or peptides such as metallothioneins bind metals to reduce their reactivity [11,12]. Other mechanisms include enzymatic transformation of metals to less toxic oxidation states (e.g., arsenate or chromate reduction), biofilm formation, and cell wall adsorption, all of which reduce intracellular metal accumulation and intensify bacterial survival in contaminated environments [13,14].

Globally, interest has increased towards using plant growth-promoting bacteria (PGPB) to not only promote plant growth but also decrease the use of fertilizer to prevent subsequent contamination of the environment as much as possible [15,16]. PGPB were shown to stimulate plant growth by synthesizing phytohormones, solubilizing phosphate and silicate, fixing nitrogen, and synthesizing volatile compounds, exopolysaccharides, and siderophores, activating antioxidant pathways [17,18]. Among these phytohormones, Indole-3-acetic acid (IAA) has been shown to be synthesized in plants naturally or by tryptophan-dependent or tryptophan-independent pathways [19]. Some Serratia species have been known for plant growth-promoting bacteria (PGPB), having a positive impact by increasing plant nutrition, reducing stress, and enhancing overall crop yield [20,21,22,23]. Recent experiments on lead removal from an aqueous medium using *Serratia marcescens CCMA 1010*, conducted regarding the expression of the *zntR* gene, which encodes ZntR, the regulator of the ZntA efflux pump of metals like Pb^2+^, Zn^2+^, and Cd^2+^ [24]. Our research group has isolated many bacterial strains from time to time from the Pb-Zn mine in Nanping, Fujian province; the reason for this was to look for Pb-Zn resistance bacteria carrying resistance genes, as well as PGP bacteria characteristics of these isolated bacteria, which could be used in the future for phytoremediation and contaminated soil restoration. We have isolated *Serratia ureilytica T6* which is a rod-shaped Gram-negative bacterium, a facultatively anaerobic bacterium of Enterobacteriaceae. Members of this genus can be commonly found in soil, water, plant rhizosphere soils, as well as polluted environments. T6 is a salt-resistant, metalloid-resistant bacterium with PGPB characteristics. The hypotheses and objective of this study are (1) to isolate and identify Pb-Zn resistant bacteria with plant growth-promoting traits, (2) to perform genomic analysis of S. *ureilytica T6* and evaluate the heavy metal tolerance ability, (3) to assess the effects of S. *ureilytica T6* on rice growth and antioxidant enzyme activities (CAT, SOD, POD, and MDA) under different levels of Pb and Zn, and (4) to determine the phytoremediation potential of *S. ureilytica T6*. Thus, this study obtained a complete genome sequence analysis of *Serratia ureilytica T6*, and genes encoding multiple heavy metal-resistant determinants were identified along with average nucleotide identity (ANI) analysis. We also identified several genes encoding potential plant growth-promotion determinants and investigated the ability of *Serratia ureilytica* T6 for promoting rice growth in heavy metal-contaminated environments.

## 2. Materials and Methods

### 2.1. Strain Isolation and Identification

Soil samples were collected from a rice field downstream of the Bayangwai lead–zinc mine in Nanya Town, Jianou, Nanping City, Fujian Province, at a depth of about 5–15 cm. 1 g of soil sample were suspended into 9 mL of sterile water, shaken at 180 rpm for 30 min, let stand for 5 min, and then the supernatant was spread onto R2A agar medium containing different heavy metal concentrations (2 mmol, 4 mmol). Then, the different suspensions were incubated at 28 °C for 24–48 h [25]. After colonies had emerged, well-growing bacterial colonies with different phenotypes were selected based on their morphology and color. Then, a single colony was purified multiple times to obtain single bacterial isolates. The selected strains were stored at −80 °C after growth in R_2_A and addition of glycerol with a final concentration of 15% glycerol. DNA of each strain was extracted according to the method indicated in the DNA extraction kit (Kit: Bacterial Genomic DNA Extraction Kit from Tiangen Company, Beijing, China). Media used in the experiment have detailed information in Supplementary Table S2.

### 2.2. Genotypic Analysis of Strain T6

#### 2.2.1. Amplification of Conserved 16S Ribosomal RNA Gene Sequences and Construction of a Phylogenetic Tree Based on 16S rRNA Gene

Using total bacterial DNA as a template, a PCR system, using primers 27F/1492R (27F (5′-GTTTGATCMTGGCTCAG-3′) and 1492R (5′-TACGGYTACCTTGTTACGAC TT -3′) [26,27], was used to amplify 16S ribosomal RNA (rRNA) gene sequences, and the amplified PCR product was subsequently sequenced. PCR products were sequenced by Biosune Company (Fuzhou, China) using the Sanger method based on the EzTaxon database (http://eztaxon-e.ezbiocloud.net) accessed on 1 June 2024 [28]. Together with the existing Serratia genomes available in NCBI, a phylogenetic tree was constructed using the Neighbor-Joining (NJ) method in MEGAX softwareversion 10. Parameters were set to Kimura’s two-parameter model with a bootstrap value of 1000.

#### 2.2.2. Whole Genome Analysis and Annotation of *S. ureilytica* T6

The assembled whole-genome sequence of *S. ureilytica* T6 was submitted to the NCBI PGAAP (Prokaryotic Genome Annotation Pipeline) database for manual annotation. The annotation generated a GBK format file containing nucleotide sequences, protein sequences, and annotation information. Functional annotation and gene prediction were conducted using the RAST (Rapid Annotation using Subsystem Technology) v2.0 database (http://rast.nmpdr.org) accessed on 1 June 2024. Genes encoding functions involved in plant growth promotion were also identified using RAST. Sequence alignment analysis was performed using BLAST (https://ncbi.nlm.nih.gov/BLAST) accessed on 1 June 2024. GO (Gene Ontology) analysis was conducted with BLAST2GO software accessed on 1 June 2024, and COG (Cluster of Orthologous Groups of proteins) functional classification was performed using the WebMGA-server online tool. Detailed methodology and bioinformatics of whole-genome sequencing of T6 are provided in Supplementary File.

#### 2.2.3. Average Nucleotide Identity (ANI) Analysis

Average Nucleotide Identity (ANI) evaluates the phylogenetic relationship between species at the whole-genome level based on genome sequences. The whole genome of *S. ureilytica* T6, combined with the 20 most related type strains with genome sequence available to the public, was used to calculate ANI values through the online tool JSpeciesWS (http://jspecies.ribohost.com/jspeciesws/#analyse) accessed on 1 July 2024.

### 2.3. Phenotypic Characterization of S. ureilytica T6

Overnight cultures of strain *S. ureilytica* T6 were inoculated into 50 mL of R2A medium at 28 °C with 180 rpm shaking. After 24 h of growth with shaking, cells were centrifuged [6000 rpm, 10 min, 4 °C], treated as described below, and observed using scanning electron microscopy (SEM). Cells were harvested and washed three times with cold (4 °C) phosphate-buffered saline (0.2 M PBS, pH 7.2). Fixation was performed with 2.5% glutaraldehyde (24 h, 4 °C). Fixed cells were dehydrated through a series of alcohol dehydration steps (30%, 50%, 70%, 85%, 95%, and 100%) and finally freeze-dried and sputter-coated. The samples were then viewed using a scanning electron microscope, JSM-6390 SEM (JEOL, Tokyo, Japan). Growth of *S. ureilytica T6* was determined in R_2_A liquid medium at various times. The optimal growth temperature, pH, tolerance to salt, and motility were also determined by measuring the OD600 of the strain grown in R_2_A medium after 24 h. After growth of T6 for 48 h, the bacterial cultures were used to perform Gram staining as described earlier [29].

### 2.4. Determination of the Minimal Inhibitory Concentration

The minimal inhibitory concentration (MIC) was determined as described in a previous publication [30]. To determine the level of resistance to various metals of strain T6 and other strains were grown on R_2_A agar plates containing different Cu(II) (CuSO_4_·5H_2_O), Zn(II) (ZnSO_4_·7H_2_O), Pb(II) (Pb(NO_3_)_2_), As(III) (NaAsO_2_), Sb(III) (KSbC_4_H_4_O_7_·½H_2_O), and Cd(II) (CdCl_2_) concentrations to determine the minimal inhibitory concentration (MIC).

### 2.5. Physiological and Biochemical Characteristics of the Strain

To assess the physiological and biochemical characteristics of T6, the following parameters were determined.

#### 2.5.1. Gelatin Liquefaction Test

This test assesses if the strain is able to produce protease. Fresh seed culture was stab-inoculated into the gelatin liquefaction medium. A tube without inoculum served as the blank control. After incubation at 28 °C for 3 days, cultures were placed in a 4 °C refrigerator for 30 min. Once the control tube solidified, the inoculated tubes were observed for liquefaction. Liquefaction indicated a positive result; no liquefaction indicated a negative result [31,32].

#### 2.5.2. Catalase Test

A small amount of a colony was smeared on a clean slide, and a drop of 5% hydrogen peroxide (H_2_O_2_) was added. The presence of bubbles indicated a positive reaction; the absence of bubbles indicated a negative reaction [33].

#### 2.5.3. Tween Hydrolysis Test

Esterase secretion was assessed using the described protocol here. Fresh seed culture was inoculated onto LB solid media containing Tween 80 and Tween 20 separately. After incubation at 28 °C for 7 days, the appearance of white halos around colonies indicated a positive result; absence indicated negative [34].

#### 2.5.4. Starch Hydrolysis Test

A fresh seed culture was inoculated onto soluble starch medium and incubated at 28 °C for 24 h. Iodine solution was evenly dropped onto the plate to cover the medium surface [35]. The presence of a clear zone around the colonies indicated starch hydrolysis (amylase positive). If the medium turned dark blue, starch was not hydrolyzed (amylase negative).

#### 2.5.5. Hydrogen Sulfide (H_2_S) Production Test

Fresh seed culture was stab-inoculated into SIM (Sulfide-Indole-Motility) medium and incubated at 28 °C for 2–7 days [36]. The presence of black precipitate in the medium indicated a positive result; absence indicated negative.

#### 2.5.6. Methyl Red Test

This test assesses acid production from glucose fermentation. Fresh seed culture was inoculated into glucose-peptone broth and incubated at 28 °C, 180 rpm for 48 h. After incubation, 3–4 drops of methyl red reagent were added. A red color indicated a positive result; yellow indicated negative [35].

#### 2.5.7. Nitrate Reduction Test

This test assesses the strain’s ability to reduce nitrate. Fresh seed culture was inoculated into nitrate reduction medium and incubated at 28 °C, 180 rpm for 4 days. Then, 1 drop each of Griess reagent A and B was added to a sample of the culture [35]. A color change to pink, orange, or brown indicated the presence of nitrite and a positive result. If no red color appeared, 1–2 drops of diphenylamine reagent were added. A blue color indicated no nitrate reduction, while no blue color indicated nitrate or nitrite reduction to other compounds, also considered positive.

#### 2.5.8. Sole Nitrogen Source Test

Different nitrogen sources (ammonium sulfate, potassium nitrate, ammonium chloride, peptone, urea, L-leucine, L-methionine, L-aspartic acid, L-arginine, and DL-histidine) were added individually to the nitrogen utilization basal medium to a final concentration of 0.5–1% (*w*/*v*). Fresh seed culture was inoculated into each medium, with nitrogen-free basal medium as the negative control. Cultures were incubated at 28 °C, 180 rpm for 3 days, and growth was observed.

#### 2.5.9. Sole Carbon Source Test

Different carbon sources (glucose, beef extract, gluconic acid, trisodium citrate, arabinose, trehalose, mannose, maltose, mannitol, soluble starch, ethanol, sucrose, malic acid, glycerol) were added individually to the carbon utilization basal medium to a final concentration of 0.5–1% (*w*/*v*). Fresh seed culture was inoculated into each medium, with carbon-free basal medium as the negative control. Cultures were incubated at 28 °C, 180 rpm for 3 days, and growth was observed.

### 2.6. Screening for Plant-Growth-Promoting Activities

#### 2.6.1. IAA Generation of *S. ureilytica* T6

The fully grown bacterial cultures were inoculated in 100 mL of NB, and the mixture was then shaken vigorously for 24 h using a rotary shaker. Then, 1 mL of each bacterial culture was transferred into a new 100 mL NB with the addition of 5 mL of L-tryptophan as an IAA precursor. Concurrently, non-inoculated control sets were prepared. The 1.5 mL bacterial cultures were transferred to Eppendorf tubes and centrifuged for 7 min at 7000 rpm [37]. The IAA concentration was determined using a spectrophotometer with a wavelength of 535 nm, and the IAA concentration of each isolate was compared to the IAA standard curve.

#### 2.6.2. Phosphate Solubilization by *S. ureilytica* T6

The isolates were cultured for 72 h at 28 °C using nutrient broth to determine their phosphate solubilization ability. Then, they were inoculated on phosphate (organic and inorganic) growth medium for 24–48 h. The halo zone formation indicated a phosphate solubilization ability [38].

#### 2.6.3. Determination of Siderophore Production of *S. ureilytica* T6

On Chrome Azurol S (CAS) agar, the ability of *S. ureilytica* T6 to produce siderophore was assessed. Strain T6 was spot-injected on CAS agar and incubated for 72 h at 33 °C. The formation of an orange halo zone around the bacterial colony indicated a positive result, as the siderophores removed the Fe from the Fe–CAS dye complex in the media [39,40,41].

#### 2.6.4. Potassium Solubilization Assay for *S. ureilytica* T6

A quantitative estimation of potassium solubilization for T1–T14 strains was determined by using flame photometry. In 100 mL of sterilized Aleksandrov broth bearing mica as a mineral potassium source in a 250 mL Erlenmeyer flask, 1% of bacterial suspension was inoculated and incubated at 28 °C for 10 days. Flasks were inoculated on a rotary shaker at 30 °C at 150 rpm, and after 7, 14, and 21 days, each flask was checked for potassium release by flame photometry as per the method outlined by [42].

#### 2.6.5. ACC Deaminase Activity

The previously described method by Penrose and Glick [43] was followed to estimate the ACC Deaminase activity of the isolates. Briefly, 100 µL of an overnight culture of each isolate was transferred to sterilized tubes containing 5 mL of half-strength TSB medium and incubated at 28 °C for 48 h in a rotary shaker, using constant agitation at 160 rpm. A loopful of each bacterial isolate growing in liquid culture was streaked into a DF minimal salts agar plate containing freshly made 3 mM ACC solution. Plates were incubated at 28 °C for almost a week. The presence of growth in the ACC-DF agar plates was considered positive. The experiment was conducted three times.

### 2.7. T6 Effected Growth of Rice Seedlings Under Lead and Zinc Stress

A hydroponic experiment was conducted using ‘Yongyou No. 9’ as a rice variety, and inoculation was performed with *Serratia ureilytica* T6. The nutrient solution used was ½-strength Hoagland nutrient solution with pH = 5.6 contained 0.505 mM KNO_3_, 0.910 mM MnCl_2_·4H_2_O, 0.150 mM Ca(NO_3_)2·4H_2_O, 0.1 mM NH_4_H_2_PO_4_, 0.1 mM MgSO_4_·7H_2_O, 0.060 mM H_2_MoO_4_·H_2_O, 4.630 mM H_3_BO_3_, 0.030 mM CuSO_4_·5H_2_O, 0.160 mM ZnSO_4_·7H_2_O, 1.640 mM FeSO_4_·7H_2_O, and 0.810 mM NA_2_–EDTA. The installed climate chamber was set to a daily photoperiod of 16 h at 26–27 °C with 230 µmol m^−2^ s^−1^ photon flux, followed by a night period of 8 h at 24 °C. Plants were grown for 6–7 weeks, while nutrient solutions were changed twice a week. The experiment was designed to have three treatments at three levels, namely: control (Ck) as; 50Pb, 100Pb, 150Pb, 50Zn, 100Zn, 150Zn, stress treatment (50, 100, 150 mg kg^−1^ Pb(Ⅱ)) and (50, 100, 150 mg kg^−1^ Zn(Ⅱ)), mitigation treatment (Pb^2+^; 50, 100, 150 mg kg−1 Pb(Ⅱ) +T6) and (Zn^2+^; 50, 100, 150 mg kg^−1^ Zn(Ⅱ)+T6). Strain T6 was inoculated into LB liquid medium and cultured at 28 °C, 180 rpm, and cells were collected through centrifugation at OD600 = 1.

Rice seeds were surface sterilized with 0.5% NaClO for 6 min, then thoroughly rinsed with sterile water multiple times. The disinfected seeds were soaked in clean water at a constant temperature (28 °C) until germination (radicle emergence). They were then transferred onto black plastic mesh and germinated in the dark. After the shoots turned green, the seedlings were exposed to light and transferred to ½-strength Hoagland nutrient solution. There were three replicates for each treatment.

During transplantation, uniformly growing seedlings were selected and transferred (two seedlings per hole) into black plastic pots (23.3 cm × 16.5 cm × 10.5 cm), with eight holes per pot. Sponges were used to fix the seedlings upright. After transplantation, a bacterial suspension was added to the nutrient solution at a 0.25% inoculation volume. After 24 h, Pb and Zn solutions were added at the corresponding concentrations. Plants were cultured for 7 days, after which they were harvested, and data were recorded.

### 2.8. Net Photosynthetic Rate and Chlorophyll Fluorescence Determination

Chlorophyll fluorescence parameters of rice seedlings were measured using a Hansatech Pocket PEA Chlorophyll Fluorimeter. Before measurement, the seedlings were dark-adapted for 20 min, after which the potential activity of PSII (Fv/Fo) and the maximum photochemical quantum efficiency of PSII (Fv/Fm) were determined.

### 2.9. Measurement of Rice Seedling Growth Parameters

After harvesting, six rice seedlings were randomly selected from each pot and divided into shoots and roots. The length of each part was measured and averaged. The fresh weight (FW) of each seedling was determined using an analytical balance. The samples were then placed in an oven and heated at 105 °C for 120 min to inactivate enzymes, followed by drying at 80 °C to a constant weight. The dry weight (DW) was then measured with an analytical balance.

### 2.10. Determination of Antioxidant Enzymes in Rice Seedlings

Frozen rice leaf samples (0.5 g) were homogenized in 10 mL of phosphate-buffered solution (PBS, pH 7.8) and then centrifuged at 10,000 rpm for 20 min at 4 °C. The supernatant was collected for the determination of SOD, CAT, POD, and MDA activities. SOD activity was determined according to the method described by Lu et al. [44]. POD activity was measured following the method of Kenawy et al. [45], recording the absorbance change in the supernatant at 470 nm every 30 s for a total of 4 readings. CAT activity was determined based on the method of Basilio-Apolinar et al. [46], measuring the absorbance of the supernatant at 240 nm every 30 s for 4 readings. Determination of malondialdehyde (MDA) was conducted using the thiobarbituric acid method, earlier used by Chen et al., 2022 [47].

### 2.11. Determination of the Heavy Metal Content

Rice seedlings were divided into shoots and roots. After drying, 0.1 g of each sample was placed in a PTFE crucible, followed by the addition of 5 mL HNO_3_ (65%) and 2 mL H_2_O (70–72%). Samples were digested, diluted with ultrapure water, and made up to 30 g. The solutions were filtered through a 0.22 μm PES membrane and analyzed for metal content using Inductively Coupled Plasma–Mass Spectrometry (ICP–MS).

### 2.12. Data Processing and Analysis

Data were analyzed using IBM SPSS Statistics 21.0, and Least Significant Difference (LSD) tests were performed to evaluate treatment effects after performing the ANOVA as its more sensitive than other test to detect differences within treatments. Graphs were plotted using Origin 2021 software.

## 3. Results

### 3.1. Isolation and Characterization of Heavy Metal-Resistant Bacteria

Different bacterial strains were isolated from the soil of the lead–zinc mine by selection for the ability to withstand high concentrations of various metals, including zinc and lead. The soil samples were collected up to 15–20 cm, the soil characteristics were recorded from a soil sample prior to bacteria isolation, and key nutrients recorded were total nitrogen (3.1 g/kg), total phosphorus (0.56 g/kg), total potassium (1.21 g/kg), and organic matter (2.3%). The PH of the soil was 5.1. There were 14 strains isolated (T1–T14), cultured on R_2_A plates, and able to withstand high concentrations of heavy metals (Pb(Ⅱ), Zn(Ⅱ), Cd(Ⅱ), Cu(Ⅱ), As(Ⅲ), and Sb(Ⅲ). Pure cultures were obtained, followed by DNA extraction and subsequent Sanger sequencing for 16S rRNA gene analysis. The sequence information of the 14 strains was analyzed on the EZBioCloud database. The 14 strains were divided into 5 genera and included 2 strains of Bacillus, 1 strain of Serratia, 2 strains of Chryseobacterium, and 1 strain of LysiniBacillus. The remaining eight strains belonged to the genus Burkholderia (Table 1).

**Figure 1:**
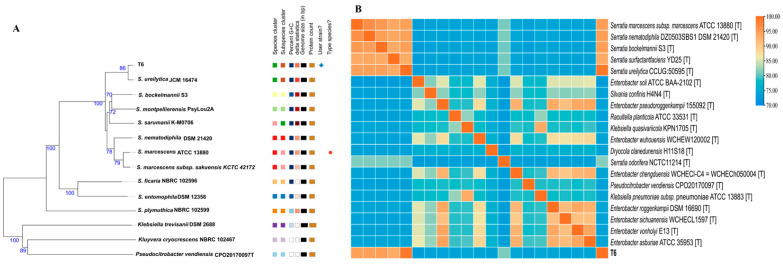
(**A**) The system evolution tree of the *S. ureilytica* T6. Bootstrap percentages greater than 50% are shown at branching points, which are based on 1000 replications. Bar 0.050 substitutions per nucleotide position. (**B**) Genomic relationship between *S. ureilytica* T6 and other 20 most related type strains: ANI values as determined with the ANIb algorithm are shown in Supplemental Material (Table S2).

Based on whole genome sequence alignment and physiological and biochemical characteristics, strain T6 was classified within the genus Serratia. The results based on the fast genome and metagenome database through the TYGS platform accessed on 1 October 2024 (https://tygs.dsmz.de) indicated that the closest type strain is *Serratia ureilytica* JCM16474 (Figure 1A).

### 3.2. Average Nucleotide Identity (ANI) Analysis

We further determined the phylogeny through ANI analysis comparing strain T6 with 20 most related type strains with genome sequences available in public, as shown in Figure 1 (the ANI (%) values of the strains are shown in Supplementary Table S2). The results indicate that strain T6 is most closely related to *Serratia ureilytica* CCUG:50595^T^, with an ANI value of 99.07% (Table S2). Since ANI values above 96% can be used to confirm strains as belonging to the same species, strain T6 was identified as *Serratia ureilytica*. ANI calculation performed using the whole genome indicates that T6 belongs to *Serratia ureilytica*.

### 3.3. Whole-Genome Sequencing and Genome Annotation of T6

A total of 1,822,799,763 kb of raw sequencing data was obtained for strain T6. After quality trimming, 1,711,319,145 kb of clean data were generated. The assembly resulted in a single scaffold with a genome length of 5,102,941 bp and a GC content of 59.74%. The genome consists of only one chromosome, which has a circular structure. By comparing genes against databases for annotation, the functions and related descriptive information of genes were identified, providing an overall functional classification of the gene set. The genome of *Serratia ureilytica* T6 was submitted to NCBI under Biosample ID (SAMN18104335), Bioproject ID (PRJNA224116), and single complete chromosome (CP071320.1). The details of T6 genome can be found in NCBI using the link; https://www.ncbi.nlm.nih.gov/nuccore/CP071320.1, accessed on 1 October 2024.

### 3.4. GO Database Annotation

**Figure 2:**
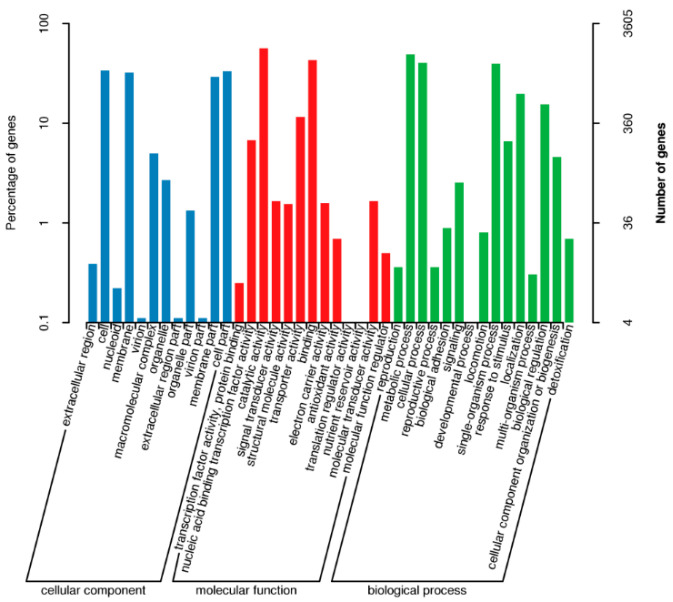
GO Function Annotation Distribution Map of *S. ureilytica* T6. Left side scale showing percentage of genes, right side scale showing number of genes involved in cellular components, molecular function, and biological process.

The GO (Gene Ontology) functional annotation of *S. ureilytica* T6 genes is shown in Figure 2. Among the GO annotation results, 4531 entries are related to molecular function, accounting for approximately 23% of the total; 8600 entries are related to cellular components, about 43.3% of the total; and 6553 entries are related to biological processes, making up about 33.3% of the total. GO analysis was performed to identify the biological functions associated with the differentially expressed genes in different biological processes, molecular functions, and cellular components as shown in GO classification histogram.

### 3.5. COG Database Annotation

**Figure 3:**
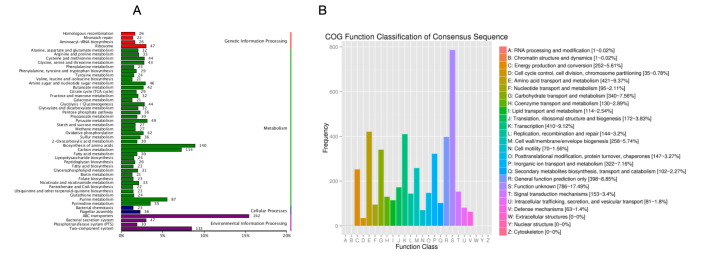
COG function annotation distribution map of *S. ureilytica* T6. Number of annotated genes and biological functions (**A**), functional classification of genes (**B**).

COG (Cluster of Orthologous Groups of proteins) is a database that collects clusters of orthologous genes, which are assumed to have descended from a common ancestral protein and share the same function. The database classifies proteins into 26 categories. By comparison, a protein sequence can be annotated into a specific COG category, allowing the prediction of the sequence’s function. The COG functional annotation of *S. ureilytica* T6 genes is shown in Figure 3A. A total of 6171 protein-coding genes of strain T6 were classified in the COG database, while 1395 genes remained unclassified. According to Figure 3B, the largest group of genes in strain T6 is those related to function prediction, totaling 828 genes. The next largest groups are genes related to transcription (784 genes) and carbohydrate transport and metabolism (458 genes), indicating that the primary role of the encoded functional proteins is to maintain cellular life and genetic metabolism.

Genes encoding functions correlated to RNA processing and modification are the fewest, with only one gene. There are 70 genes encoding functions associated with cell motility, consistent with the motile phenotype observed in *S. ureilytica* T6. Additionally, there are 252 genes involved in energy production and conversion, 63 genes correlated to defense mechanisms, and 153 genes linked to signal transduction mechanisms.

### 3.6. RAST System Annotation

**Figure 4:**
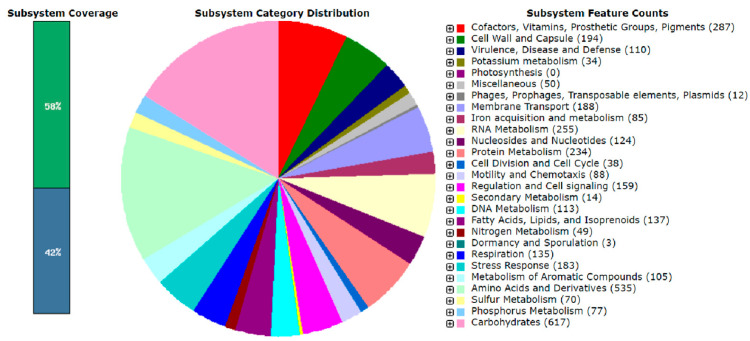
Subsystem category distribution of the whole genome of *S. ureilytica* T6. Bar on the left indicates 58% (coding sequences) were assigned to known subsystems, while 42% (unclassified) were under any defined functional category.

After annotation of the genome of *S. ureilytica* T6 using the RAST system, the distribution of gene subsystem categories is shown in Figure 4. The annotation results indicate that the genome size of strain T6 is 5,102,941 bp with a G + C content of 59.7%. A total of 4712 genes were annotated in the genome of *S. ureilytica* T6. Among these, a large proportion of genes are related to amino acids and their derivatives (535 genes) and carbohydrates (617 genes), indicating that the primary function of the proteins encoded by the genome is to maintain cellular life and genetic metabolism.

There were 110 genes encoding functions correlated to virulence, disease, and defense, with heavy metal resistance classified under this subsystem. Additionally, there are 49 genes encoding functions related to nitrogen metabolism, 77 genes related to phosphorus metabolism, 34 genes related to potassium metabolism, 85 genes encoding functions involved in iron acquisition and metabolism, and 14 genes associated with secondary metabolism. These functions are correlated to plant growth promotion. Through RAST genome annotation analysis, multiple genes encoding functions correlated to lead (Pb) and zinc (Zn) resistance were identified in the genome of *Serratia ureilytica T6*. No *pbr* operon was found in the T6 genome; however, three genes encoding putative Zn, Cd, Pb transporting P-type ATPase were identified. Genes associated with PGP were also observed in T6. Strain T6 is able to solubilize both organic and inorganic phosphorus. The main mechanism for phosphate solubilization in bacteria is the synthesis and secretion of gluconic acid. The production of gluconic acid requires glucose dehydrogenase and its cofactor PQQ. Genes encoding PQQ-dependent glucose dehydrogenase and the operon composed of *pqqABCDEF*, which is necessary for PQQ biosynthesis, were found in the genome of T6. Genes encoding functions correlated to citrate synthesis were also identified: citrate synthase (EC 2.3.3.1) and aconitate hydratase (EC 4.2.1.3). Citrate has been repeatedly reported to be correlated with the phosphate-solubilizing function of growth-promoting bacteria. For IAA production, the genome of T6 contains *ipdC* encoding indole-3-pyruvate decarboxylase and *dhaS* encoding indole-3-acetaldehyde dehydrogenase. Both gene products are required for the conversion of tryptophan to IAA.

### 3.7. Morphological Characteristics of S. ureilytica T6

**Figure 5:**
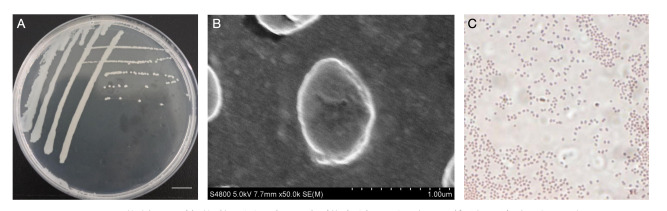
Colony morphology of the bacterial isolate grown on nutrient agar, showing smooth, circular, and creamy-white colonies (**A**). Scanning electron micrograph (SEM) revealing rod-shaped bacterial cells with smooth surfaces (**B**). Gram staining of the isolate observed under a light microscope, showing Gram-negative short rods arranged singly or in clusters (**C**). Scale bar for (**A**) is 3 mm, (**B**) is 1.00 μm and (**C**) is 2 μm.

The single colony morphology of T6 on *S. ureilytica* LB solid medium is shown in Figure 5. Colonies were opaque, milky white, circular, with raised, smooth, moist surfaces, and even edges, without obvious pigment production. Further, we used scanning electron microscopy for strain T6, which revealed nearly spherical short rod shapes, without flagella or spores. After Gram staining, observation under an optical microscope showed the cells stained red, indicating that strain T6 is Gram-negative.

### 3.8. Growth Characteristics of S. ureilytica T6

**Figure 6:**
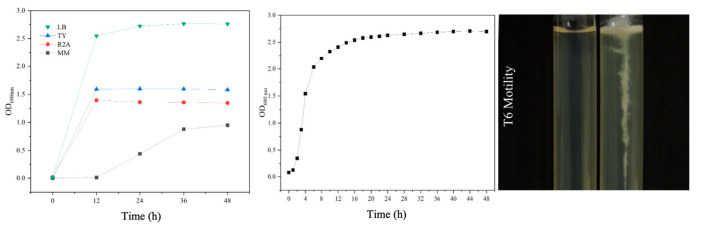
Growth characteristics of *S. ureilytica* T6. Growth of *S. ureilytica* T6 in different media, LB, TY, R_2_A, and MM (**left**). Growth curve on LB medium with optical density (OD_600_) (**middle**) and motility assay on semisolid medium puncture test of T6 (**right**).

The growth of *S. ureilytica* T6 in different media is shown in Figure 6 (left). *S. ureilytica* T6 was able to grow in all four tested media: LB, TY, R_2_A, and MM. Among these, strain T6 showed the best growth in LB medium, followed by TY and R_2_A media, while T6 grew slowly in MM medium. Therefore, LB medium was determined to be the optimal growth medium for strain T6. A growth curve was obtained (Figure 6. (middle)) in LB liquid medium at 28 °C and 180 rpm. *S. ureilytica* T6 has a very short lag phase and enters the logarithmic phase after just 1 h, during which the bacteria begin to multiply rapidly. After 18 h, it reached the stationary phase, which lasted for a relatively long time. The semi-solid stab test was used to observe the motility of *S. ureilytica* T6 (Figure 6 (right)). In semi-solid agar, *S. ureilytica* T6 grew in a spreading pattern outward from the stab line, indicating that *S. ureilytica* T6 is motile.

### 3.9. Environmental Factors Influencing S. ureilytica T6

Temperature is the most direct environmental factor affecting microbial life activities, and microorganisms are highly sensitive to temperature. The growth performance of *S. ureilytica* T6 follows the order: 28 °C > 37 °C > 18 °C. Therefore, the optimal growth temperature for strain T6 is 28 °C with a wide range of 18–37 °C. Microorganisms can grow over a wide pH range of 2–8, but the vast majority live between pH 5.0 and 9.0. Our experimental results revealed that *S. ureilytica* T6 can grow in a range of pH 4.08–9.51.

### 3.10. Minimum Inhibitory Concentration of Heavy Metal(loid)s for T6

To determine the heavy metal resistance level of strain T6, the MICs of six heavy metal ions were measured using the inorganic salt (MM) basal medium. The results indicate that strain T6 has a high resistance to Zn, with a minimum inhibitory concentration of 9 mmol·L^−1^, and also shows certain tolerance to other heavy metal ions as; Pb(Ⅱ) (1.5 mmol·L^−1^), Cd(Ⅱ) (2.7 mmol·L^−1^), Cu(II) (0.6 mmol·L^−1^), As(III) (7.0 mmol·L^−1^), and Sb(III) (3.0 mmol·L^−1^). This suggests that the strain possesses broad-spectrum resistance to multiple heavy metals; details of T6 heavy metal-resistance determinants are provided in Supplementary Table S4.

### 3.11. Physiological and Biochemical Characteristics of S. ureilytica T6

#### 3.11.1. Enzymatic Activity Tests

Different physiological and biochemical tests were carried out for T6. The enzymatic activity tests include Catalase, Gelatin Liquefaction, Starch Hydrolysis, and Tween 20, 80 Hydrolysis tests. T6 contained catalase, where H_2_O_2_ breaks down by catalase (bubbles). Gelatin Liquefaction was observed as T6 produces and can degrade gelatin. Tween 20 and 80 hydrolysis was observed in T6 as it secretes esterase, while T6 was unable to degrade starch as it did not produce amylase.

#### 3.11.2. Metabolic Capability Tests

Metabolic capabilities of T6 were tested as the nitrate reduction test, Hydrogen Sulfide (H_2_S) Production test, and methyl red production test. T6 does not have the ability to ferment glucose to produce large amounts of acids such as pyruvic acid. T6 has the ability to reduce nitrate to nitrite, but we did not observe T6 producing, and it did not have the ability to ferment mixed acids.

#### 3.11.3. Nitrogen Source Utilization

*S. ureilytica* T6 was capable of utilizing a wide range of nitrogen sources as the sole nitrogen source including inorganic sources (Ammonium sulfate, potassium nitrate, ammonium chloride, urea) and organic sources (Peptone, L-leucine, L-methionine, L-aspartic acid, L-arginine, DL-histidine). This indicates that *S. ureilytica* T6 has versatile nitrogen assimilation ability.

#### 3.11.4. Carbon Source Utilization

*S. ureilytica* T6 grows well on the following carbon sources, showing a broad metabolic flexibility: glucose, gluconic acid, beef extract, trehalose, arabinose, trisodium citrate, mannose, maltose, mannitol, sucrose, soluble starch, glycerol, and malic acid. This demonstrates T6’s ability to metabolize sugars, organic acids, and polysaccharides, but not ethanol.

### 3.12. Plant Growth-Promoting Characteristics of T6

#### 3.12.1. IAA Production Characteristic

The Salkowski colorimetric method was used to rapidly determine the IAA production capacity of the bacteria. As shown in Table 2, with the exception of strains T2 and T8, all other strains were able to convert tryptophan into indole acetic acid.

Through growth and metabolism, resulting in the culture medium turning red under the action of the Salkowski colorimetric reagent, indicating their IAA production. Strain T6 exhibited the strongest IAA production capacity, reaching a yield of 26.36 mg·L^−1^, significantly higher than that of the other strains (Table 2).

**Table 2:** The ability to solubilize phosphorus and potassium, generate siderophores, and IAA production by different strains. Diameter of the clear zone (D) to the diameter of the colony (d) (D/d).

Strains	Phosphorus Solubilization (Organic) (D/d)	Phosphorus Solubilization (Inorganic) (D/d)	Potassium Solubility (D/d)	Siderophores Production (D/d)	IAA Production mg·L^−1^
T1	2.19 ± 0.050 ef	1.17 ± 0.000 b	2.75 ± 0.140 e	1.77 ± 0.180 e	10.02 ± 0.110 g
T2	-	-	-	-	1.01 ± 0.212 i
T3	2.36 ± 0.180 e	1.2 ± 0.002 b	2.72 ± 0.100 e	1.58 ± 0.070 e	7.012 ± 0.332 h
T4	3.60 ± 0.300 bc	1.17 ± 0.000 b	3.44 ± 0.220 ab	3.21 ± 0.190 c	21.21 ± 0.421 c
T5	3.92 ± 0.070 a	1.36 ± 0.016 b	3.57 ± 0.060 a	3.58 ± 0.140 b	18.78 ± 0.325 d
T6	2.37 ± 0.080 e	1.17 ± 0.000 b	2.91 ± 0.160 e	2.23 ± 0.430 d	26.36 ± 0.21 a
T7	3.77 ± 0.110 ab	1.27 ± 0.000 b	3.47 ± 0.140 ab	4.18 ± 0.340 a	19.61 ± 0.231 c
T8	-	-	-	-	0.97 ± 0.421 i
T9	2.68 ± 0.060 d	-	-	-	23.52 ± 0.234 b
T10	1.70 ± 0.110 g	1.31 ± 0.001 b	3.14 ± 0.120 cd	1.75 ± 0.00 e	13.27 ± 0.182 f
T11	-	-	-	-	13.87 ± 0.041 f
T12	2.74 ± 0.040 d	-	-	-	21.61 ± 0.01 b
T13	2.14 ± 0.260 f	1.25 ± 0.014 b	3.1 ± 0.050 d	2.32 ± 0.480 d	13.09 ± 0.032 f
T14	3.44 ± 0.000 c	1.61 ± 0.008 a	3.32 ± 0.250 bc	3.97 ± 0.060 a	16.28 ± 0.421 e

Note: Letters (a–i) shows significance level.

#### 3.12.2. Phosphate-Solubilizing, Potassium-Releasing, and Siderophore-Producing and ACC Deaminase Activity

The growth status of the strains in the culture medium was observed. The size of the clear zones formed around each colony on the medium and the ratio of the diameter of the clear zone (D) to the diameter of the colony (d) (D/d) were used to preliminarily determine the phosphate-solubilizing and potassium-releasing abilities of the strains. A total of 11 strains have the ability to solubilize organic phosphate, among which strain T5 has the highest ratio of 3.44. Eight strains had the ability to solubilize inorganic phosphate and release potassium, with T14 and T5 showing the strongest abilities, having D/d ratios of 1.61 and 3.57, respectively (Table 2).

On the general CAS agar plate, the siderophores produced by the bacterial cells bind with the blue complex dye composed of CAS, HDTMA, and iron ions, changing the color from blue to orange or reddish-orange, thus generating a distinct siderophore halo. As shown in Table 2, strains T2, T8, T9, T11, and T12 do not produce orange halos and therefore lack siderophore-producing ability. Among them, strains T7 and T14 have the strongest siderophore-producing abilities, with D/d ratios of 4.18 and 3.97, which are significantly higher than those of the other strains.

**Figure 7:**
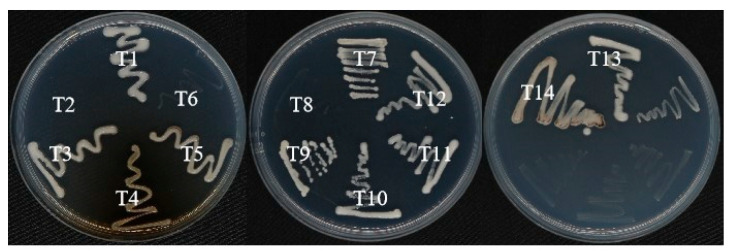
Plates showing ACC Deaminase activity of studied strains (T1–T14) on 3 mM ACC-DF media incubated at 28 °C for a week.

ACC deaminase activity for the examined strains was studied, showing that T2, T6, and T8 lacked ACC deaminase activity, while the rest of the strains displayed ACC deaminase activity, as shown in Figure 7.

### 3.13. Effect of S. ureilytica T6 Inoculation on Chlorophyll Fluorescence Parameters of Rice Leaves Under Lead and Zinc Exposure

A large number of studies have shown that heavy metal pollution is able to reduce the Fv/Fm and Fv/Fo values of plant PSII. Here we observed a slight increase in the Fv/Fm and Fv/Fo ratios between the CK (control) and the CK + mitigation treatment (Table 3), indicating that the application of *S. ureilytica T6* has a small but significant effect to protect the photochemical conversion efficiency of PSII in rice leaves. Under Pb stress, the Fv/Fm and Fv/Fo ratios of rice leaves were significantly higher than those of the control (*p* < 0.05). When the Pb stress levels were 50 mg·L^−1^ and 100 mg·L^−1^, the Fv/Fm values reached the highest level (0.8), showing an increase of 12.68% compared with the control. When the Pb stress was 150 mg·L^−1^, the Fv/Fo value reached its maximum, 41.6% higher than the control.

**Table 3:** Effect of *S. ureilytica* T6 inoculation on chlorophyll fluorescence parameters of rice leaves under Pb stress. + sign shows inoculation with T6.

Treatment	Fv/Fm	Fv/Fo
CK	0.71 ± 0.030 d	2.50 ± 0.293 d
CK+	0.72 ± 0.040 d	2.61 ± 0.401 d
50Pb	0.80 ± 0.010 b	4.05 ± 0.194 b
50Pb+	0.80 ± 0.000 ab	4.11 ± 0.093 b
100Pb	0.80 ± 0.010 b	4.05 ± 0.145 b
100Pb+	0.82 ± 0.010 a	4.57 ± 0.211 a
150Pb	0.78 ± 0.020 c	3.54 ± 0.372 c
150Pb+	0.80 ± 0.020 b	3.97 ± 0.478 b

Note: Letters (a–d) shows significance level.

Under Zn stress, the Fv/Fm and Fv/Fo ratios of rice leaves were significantly higher than those of the control (*p* < 0.05) (Table 4). However, with an increase in Zn concentration and the application of the T6 bacterial suspension, there were no significant differences in the Fv/Fm and Fv/Fo values.

**Table 4:** Effects of strain T6 on chlorophyll fluorescence parameters of rice leaves under Zn stress. + sign shows inoculation with *S. ureilytica* T6.

Treatments	Fv/Fm	Fv/Fo
CK	0.71 ± 0.025 b	2.50 ± 0.293 c
CK+	0.72 ± 0.037 b	2.61 ± 0.401 c
50Zn	0.80 ± 0.013 a	4.02 ± 0.339 ab
50Zn+	0.81 ± 0.009 a	4.28 ± 0.242 a
100Zn	0.80 ± 0.023 a	4.05 ± 0.535 ab
100Zn+	0.80 ± 0.018 a	4.10 ± 0.441 ab
150Zn	0.79 ± 0.036 a	3.82 ± 0.656 b
150Zn+	0.80 ± 0.015 a	4.05 ± 0.341 ab

Note: Letters (a–c) shows significance level

### 3.14. Effect of S. ureilytica T6 Inoculation on Growth Parameters of Rice Seedlings Under Lead Exposure

Here we observed that with increasing Pb concentration, the fresh weight, dry weight, plant height, and root length of rice showed a trend of first increasing and then decreasing. Under the 50 mg·L^−1^ Pb treatment, the above-ground biomass of rice showed no significant difference compared with the control (CK), but the fresh and dry weights of the above-ground parts were significantly higher than those of the CK treatment (*p* < 0.05), while the root length was 1.21% lower than CK. Under the 150 mg·L^−1^ Pb treatment, the biomass of rice was the lowest, significantly lower than under the other treatments (*p* < 0.05) (Table 5). The application of *S. ureilytica* T6 alleviated the damage to rice roots caused by high Pb concentrations. When the Pb concentration reached 150 mg·L^−1^, the addition of T6 bacterial suspension significantly increased the fresh weight, dry weight, and root length of the underground parts compared with the stress treatment (*p* < 0.05), with increases of 63.16%, 100%, and 17.19%, respectively (Table 5).

**Table 5:** Effect of *S. ureilytica* T6 on the growth characteristics under Pb exposure. + sign shows inoculation with T6.

	Treatment	Fresh Weight (g)	Dry Weight (g)	Length (cm)
Above Ground Part	CK	0.70.14 ± 0.145 a	0.23 ± 0.049 d	35.12 ± 1.023 a
	CK+	1.16 ± 0.133 a	0.26 ± 0.029 cd	39.99 ± 1.077 a
	50Pb	1.12 ± 0.052 a	0.33 ± 0.076 ab	39.62 ± 0.954 a
	50Pb+	1.13 ± 0.120 a	0.38 ± 0.026 a	39.95 ± 1.264 a
	100Pb	0.76 ± 0.184 cd	0.26 ± 0.041 cd	35.23 ± 1.742 b
	100Pb+	0.90 ± 0.251 bc	0.30 ± 0.027 bc	35.52 ± 1.645 b
	150Pb	0.46 ± 0.084 e	0.09 ± 0.016 e	25.66 ± 1.505 c
	150Pb+	0.61 ± 0.153 de	0.10 ± 0.015 e	32.08 ± 3.869 d
Below Ground Part	CK	0.48 ± 0.117 bc	0.08 ± 0.014 c	11.69 ± 0.940 a
	CK+	0.62 ± 0.096 b	0.09 ± 0.010 c	16.38 ± 1.285 a
	50Pb	0.73 ± 0.077 a	0.13 ± 0.019 a	14.50 ± 0.707 b
	50Pb+	0.82 ± 0.055 a	0.14 ± 0.015 a	15.65 ± 0.855 a
	100Pb	0.44 ± 0.085 d	0.11 ± 0.012 b	11.72 ± 0.733 c
	100Pb+	0.48 ± 0.119 cd	0.11 ± 0.012 b	11.87 ± 1.538 c
	150Pb	0.19 ± 0.017 f	0.03 ± 0.005 e	9.95 ± 0.677 d
	150Pb+	0.31 ± 0.038 e	0.06 ± 0.006 d	11.66 ± 0.585 c

Note: Letters (a–f) shows significance level.

### 3.15. Effect of S. ureilytica T6 Inoculation on Growth Parameters of Rice Seedlings Under Zinc Stress

With increasing Zn concentrations, the fresh weight, dry weight, and length of rice seedlings displayed a trend of first increasing and then decreasing (Table 6). Under the 50 mg·L^−1^ Zn treatment, rice exhibited the highest fresh weight, which was significantly higher than under other treatments. The dry weight was significantly higher than under other Zn stress treatments, and the plant height was significantly higher than under the control (CK), indicating that low concentrations of Zn promoted rice growth. Under the 150 mg·L^−1^ Zn treatment, rice biomass was the lowest, significantly lower than under other treatments (*p* < 0.05).

The application of the *S. ureilytica* T6 bacterial suspension promoted root elongation of rice under Zn stress. When the Zn concentrations were 50 mg·L^−1^ and 100 mg·L^−1^, the addition of T6 increased the root length of rice by 17.39% and 10.46%, respectively, compared with the non-inoculated treatments, and the differences were significant (*p* < 0.05). However, when the Zn stress concentration reached 150 mg·L^−1^, the application of T6 no longer promoted root elongation in rice (Table 6).

**Table 6:** Effect of *S. ureilytica* T6 inoculation on the growth characteristics under Zn stress. + sign shows inoculation with *S. ureilytica* T6.

	Treatments	Fresh Weight (g)	Dry Weight (g)	Length (cm)
Above ground Part	CK	1.14 ± 0.145 c	0.20 ± 0.014 bc	39.99 ± 1.077 b
	CK+	1.16 ± 0.133 c	0.24 ± 0.028 ab	39.63 ± 1.023 b
	50Zn	1.36 ± 0.222 ab	0.24 ± 0.014 ab	46.90 ± 1.192 a
	50Zn+	1.50 ± 0.123 a	0.20 ± 0.029 a	47.08 ± 0.376 a
	100Zn	1.32 ± 0.119 b	0.16 ± 0.033 cd	46.66 ± 1.839 a
	100Zn+	1.32 ± 0.137 b	0.21 ± 0.059 bc	47.44 ± 2.505 a
	150Zn	0.45 ± 0.066 d	0.11 ± 0.031 e	26.87 ± 1.429 c
	150Zn+	0.50 ± 0.059 d	0.14 ± 0.045 de	28.08 ± 1.647 c
Below ground Part	CK	0.58 ± 0.117 b	0.08 ± 0.014 ab	15.69 ± 0.940 b
	CK+	0.62 ± 0.096 b	0.09 ± 0.010 ab	16.38 ± 1.285 b
	50Zn	0.85 ± 0.148 a	0.10 ± 0.035 a	16.85 ± 0.774 b
	50Zn+	0.94 ± 0.085 a	0.10 ± 0.010 a	19.78 ± 1.943 a
	100Zn	0.89 ± 0.084 a	0.05 ± 0.023 cd	12.71 ± 0.929 d
	100Zn+	0.90 ± 0.036 a	0.07 ± 0.014 bc	14.04 ± 0.698 c
	150Zn	0.51 ± 0.044 b	0.03 ± 0.009 d	10.86 ± 0.838 e
	150Zn+	0.51 ± 0.101 b	0.05 ± 0.010 cd	10.87 ± 0.966 e

Note: Letters (a–e) shows significance level.

### 3.16. Effect of S. ureilytica T6 on MDA Content in Rice Roots Under Pb and Zn Stress

**Figure 8:**
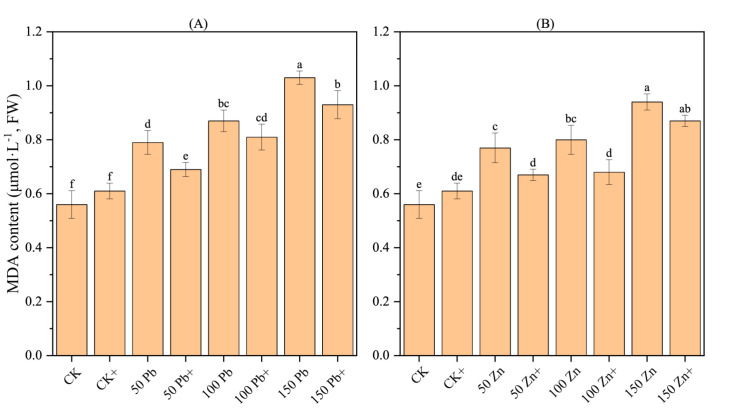
Effect of *S. ureilytica* T6 on MDA content of rice root under lead (**A**) stress and zinc (**B**) stress. + sign shows inoculation with T6. Letters (a–f) shows significance level.

Under Pb and Zn stress, the MDA content in rice roots showed an increasing trend with the rise in stress concentration compared with the control (CK), and the difference reached a significant level (*p* < 0.05) (Figure 8). This indicates that Pb and Zn stress damaged the cell membranes of rice, and the higher the concentration, the more severe the membrane lipid peroxidation. Compared with the stress treatments, the mitigation treatments (50Pb^+^, 150Pb^+^, 50Zn^+^, and 100Zn^+^) showed a decreasing trend in MDA content, with significant differences (*p* < 0.05), which were reduced by 12.99%, 15.00%, 12.66%, and 9.71%, respectively. This suggests that strain T6 was able to reduce the degree of membrane lipid peroxidation in rice to some extent, indicating that T6 alleviated Pb and Zn stress in rice.

### 3.17. Effect of S. ureilytica T6 Inoculation on SOD Activity in Rice Under Pb and Zn Exposure

**Figure 9:**
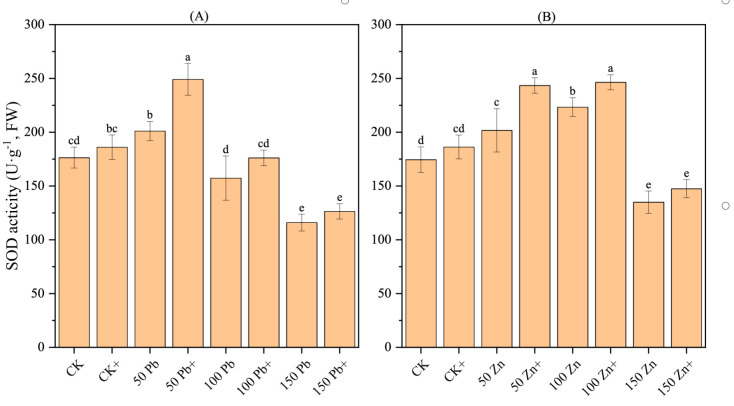
Effect of *S. ureilytica* T6 inoculation on SOD activity of rice under lead (**A**) and zinc exposure (**B**) + sign shows inoculation with *S. ureilytica* T6. Letters (a–e) shows significance level.

The SOD activity in rice exhibited a trend of first increasing and then decreasing with increasing metal stress, and the differences were significant (*p* < 0.05) (Figure 9). Under Pb stress, the 50 Pb treatment displayed the highest SOD activity, which was 13.91% higher than CK (*p* < 0.05). Under Zn stress, the 100 Zn treatment showed the highest SOD activity, 27.97% higher than CK, displaying a significant difference (*p* < 0.05). Compared with the non-inoculated treatment, the inoculated treatments (50Pb^+^, 150Pb^+^, 50Zn^+^, 100Zn^+^) showed a significant increase in SOD activity (*p* < 0.05), rising by 23.90%, 8.96%, 20.66%, and 10.37%, respectively. These results demonstrated that *S. ureilytica* T6 was able to enhance SOD activity in rice, thereby improving the plant’s antioxidant defense and alleviating Pb and Zn stress to a certain extent.

### 3.18. Effect of S. ureilytica T6 Inoculation on POD Activity in Rice Under Pb and Zn Exposure

**Figure 10:**
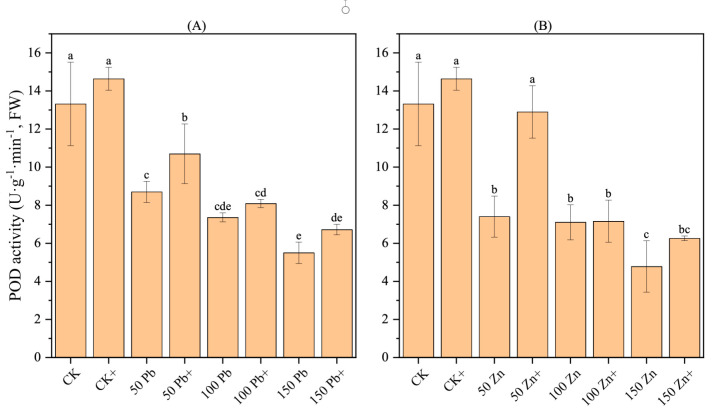
Effect of *S. ureilytica* T6 inoculation on CAT activity of rice under lead stress (**A**) and zinc stress (**B**) + sign shows inoculation with *S. ureilytica* T6. Lettters (a–e) shows significance level.

With an increase in Pb and Zn exposure, the POD (peroxidase) activity in rice showed a decreasing trend, and the differences were significant (*p* < 0.05) (Figure 10). When the concentration of Pb stress reached 150 mg·L^−1^, the POD activity was the lowest, 58.71% lower than the control (CK), showing a significant difference (*p* < 0.05). Similarly, under 150 mg·L^−1^ Zn, the POD activity reached its lowest value, 64.11% lower than CK, also with a significant difference (*p* < 0.05).

### 3.19. Effect of S. ureilytica T6 on CAT Activity in Rice Under Pb and Zn Stress

**Figure 11:**
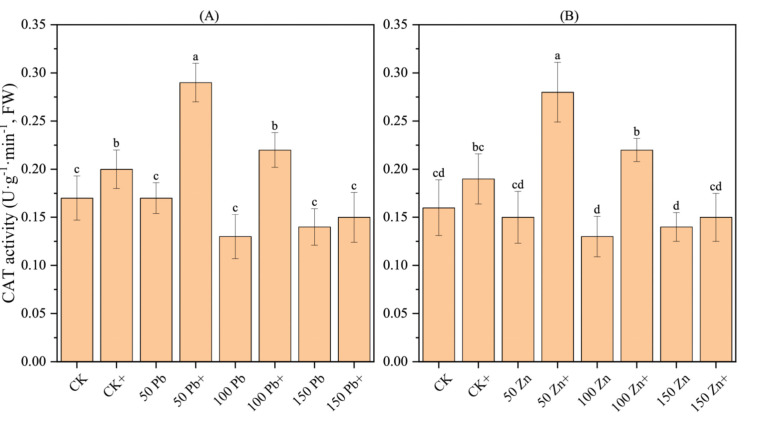
Effect of T6 on CAT activity of rice under lead (**A**) and zinc exposure (**B**) + signs show inoculation with T6. Letters (a–d) shows significance level.

With increasing Pb and Zn stress concentrations, the CAT (catalase) activity in rice displayed a trend of first decreasing and then increasing, although the differences were not significant (Figure 11). The application of *S. ureilytica* T6 bacterial suspension led to a distinct increase in CAT activity under low to moderate concentrations of Pb and Zn exposure (*p* < 0.05). At Pb concentrations of 50 mg·L^−1^ and 100 mg·L^−1^, the application of *S. ureilytica* T6 increased CAT activity by 70.59% and 69.23%, respectively, compared with the uninoculated treatment. At Zn concentrations of 50 mg·L^−1^ and 100 mg·L^−1^, CAT activity increased by 86.67% and 69.23%, respectively, after *S. ureilytica* T6 inoculation.

### 3.20. Effects of S. ureilytica T6 on Pb Content in Rice Under Pb and Zn Exposure

**Figure 12:**
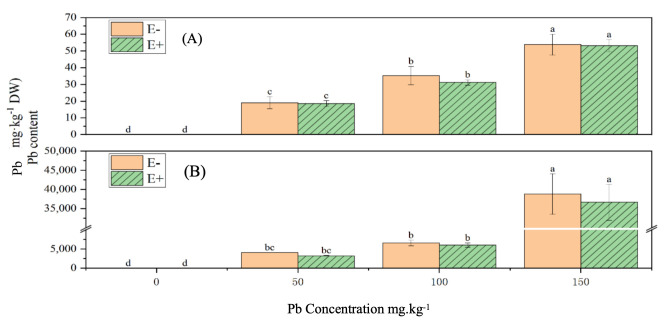
Effect of *S. ureilytica* T6 inoculation on Pb content of rice under Pb exposure. E− without inoculation, E+ inoculated with T6. (**A**): shoot; (**B**): root. Letters (a–d) shows significance level.

Under increasing Pb concentrations, the Pb content in both the shoots and roots of rice gradually increased, displaying a significant difference compared with the control group (*p* < 0.05) (Figure 12).

The application of *S. ureilytica* T6 bacterial suspension slightly reduced Pb accumulation in both the above-ground parts and roots compared with uninoculated treatments. Pb content in shoots decreased by approximately 1.25–11.69%, and in roots by 5.57–19.45%, although the differences were not statistically significant.

**Figure 13:**
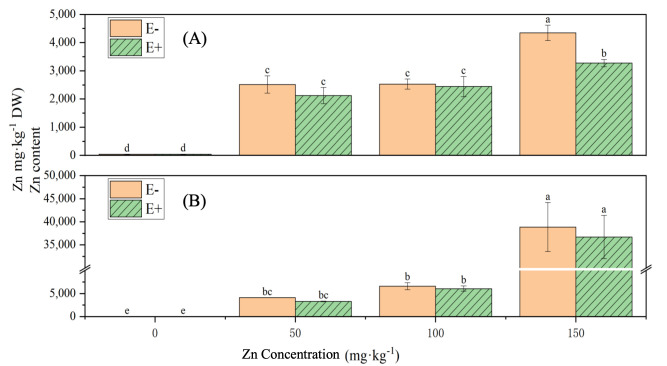
Zn accumulation in rice under Zn exposure. E- without inoculation, E+ inoculated with T6. (**A**): shoot; (**B**): root. Letters (a–e) shows significance level.

Under increasing Zn stress levels, the Zn content in both the shoots and roots of rice gradually increased, showing significant differences compared with the control group (*p* < 0.05) (Figure 13). Under 150 mg·L^−1^ Zn stress, the Zn content in the shoots of rice treated with *S. ureilytica* T6 suspension was significantly lower than that of the uninoculated treatment, displaying a 24.71% reduction. At other Zn concentrations, the Zn contents in both the shoots and roots of rice treated with *S. ureilytica* T6 were slightly lower than those in the uninoculated control, but the differences were not statistically significant.

## 4. Discussion

### 4.1. Isolation and Characterization of Heavy Metal-Resistant Bacteria

In this experiment, soil samples were collected from rice fields downstream of the Bayangwai lead–zinc mine in Nanya Town, Jianou, Nanping City, Fujian Province, China. There were 14 (T1–T14) strains isolated and identified, followed by genotypic and phenotypic characterization. We selected *Serratia ureilytica T6* because of its high heavy metal (Pb and Zn) resistance and salt tolerance. We performed whole-genome sequencing of T6 and ANI analysis, followed by genome annotation. Finally, we also explored the plant growth-promoting capabilities of T6 for possible use in future soil restoration and assisting crops to adapt and survive in diverse environments.

Serratia belongs to the bacterial genus of the order Enterobacterales [48], of which 1–2% of hospital-acquired infections (HAIs) are attributable to and able to cause outbreaks with high mortality rates [49]. According to the NCBI assortment database, the genus Serratia consists of 26 species heterogeneously dispersed in water, soil, and the rhizosphere [50]. *S. marcescens* is the most common characterized species of Serratia, followed by *S. liquefaciens*, which was first identified and classified as *Aerobacter liquefaciens* in the Enterobacter genus [49]. Here we isolated *Serratia ureilytica T6* from soil contaminated with Pb and Zn. Understanding the mechanism underlying the heavy metal resistance in T6 could provide valuable insights into bacterial adaptation to heavy metal contamination in the environment. Previously, only a few Serratia strains had been isolated from heavy metal-contaminated environments. *Serratia marcescens BM1* was isolated from maize rhizospheric soils contaminated with Cd [51]. *Serratia marcescens WZ14* was isolated from the lead–zinc–silver mine of Shanxi Province, China [52]. Here we report Pb-Zn resistant plant growth-promoting bacterial strain *Serratia ureilytica T6* displaying Pb-Zn resistance isolated from rice soils irrigated by Pb-Zn mine. On the basis of 16S sequence phylogenetic analysis, T6 was shown to be closely related to 14 other Serratia strains, while strain T6 was most closely related to *Serratia ureilytica FDAARGOS_1089* when analyzed through ANI, comparing strain T6 with thirteen other Serratia species uploaded in NCBI. ANI values are considered as best species boundary; this level of divergence is significant enough to impact subsequent taxonomic interpretations [53].

### 4.2. Whole-Genome Sequencing (WGS) and Genome Annotation and RAST Analysis

T6 has a single circular chromosome with a genome size of 5,102,941 bp with a GC content of 59.74%. To uncover the microbial diversity and in-depth study of microbes, WGS enabled researchers to unveil the metabolic capabilities of the microbial community, providing valuable insights into the microbiome. WGS facilitates the identification of functional genes for subsequent studies. Earlier, the genome size of some of the Serratia species has been documented, showing that *S. marcescens* is 5–5.6 Mb, with G + C content of 57.5–60.4%, *S. liquefaciens* 52.5–54.4%, *S. plymuthica* 53.4–56.3%, and *S. marinorubra* 53.5–58.6% [54]. Here we have a G + C content of 59.74% in T6 which is the highest among the reported Serratia strains among all enterobacteria. Three databases, GO, COG, and KEGG, were used for annotation. Here we are discussing GO and COG only in this article. The statistical summary of the gene annotation results for strain T6 is shown in Table 2. Strain T6 was also annotated using the RAST system. The GO annotation of T6 resulted in 4531 entries, of which 23% of the total were related to molecular function, while 43.3% of the total were related to cellular components, and 33.3% of the total were related to biological processes. In recent studies of *Serratia marcescens F13*, the GO function enrichment analysis revealed that the GO term mainly enriched by different genes in the three comparability groups was mostly correlated with the transport and migration of cellular or subcellular components, transmembrane transporter activity, cell localization, and flagella-dependent cell movement [55]. Based on COG analysis, the largest group of genes in strain T6 is those related to function prediction, followed by genes related to transcription, carbohydrate transport, and metabolism, indicating that the primary role of the encoded functional proteins is to maintain cellular life and genetic metabolism. We also found genes involved in energy production and conversion, defense mechanisms, and signal transduction mechanisms. These genes may provide strain T6 with more energy metabolism pathways to adapt to various environments and may play a key role in the process of metal ion efflux. The relatively high number of genes associated with signal transduction may enhance strain T6’s ability to sense environmental changes and regulate its adaptation accordingly. The role of T6 in biological processes and molecular functions suggests active metabolic versatility and enzymatic potential, which are distinctive properties of Serratia species known for their environmental adaptability and pollution resistance [22,56]. Similar genomic distribution patterns have been observed in other metal-resistant bacteria, reflecting a balance between metabolic function, stress response, and cell structure stabilization under toxic environments [57].

Through RAST genome annotation analysis, multiple genes had encoding functions correlated with lead (Pb) and zinc (Zn) resistance in the genome of *Serratia ureilytica* T6. No pbr operon was found in the T6 genome; however, three genes encoding putative Zn and Pb transporting P-type ATPase genes were identified. P-type ATPases (adenosine triphosphatases) play a critical function in transporting Pb and Zn from the inside of the cell to the outside, which is important for regulating intracellular Pb and Zn balance and resistance. P-type ATPases are transporting metal ions from the cytoplasm to the periplasm using ATP as a source of energy [24]. Additionally, eight genes (*ZhuABC*, *Zur*, *ZitB*, *MerR*, *Czc*) associated with Zn resistance and five genes encoding Zn, Cd, and Co tripartite efflux pumps were found. Since Pb and Zn ions are both divalent cations, mechanisms that confer Zn resistance might also contribute to Pb resistance. Recently it was reported that *Serratia marcescens CCMA 1010* possesses *zntR* encoding ZntR which is a known MerR-type regulator of the *ZntA* efflux pump for Zn^2+^, Pb^2+^, and Cd^2+^ [24].

Our genomic analysis provides a basis for the strong Zn-Pb tolerance in T6. The existence of multiple P-type ATPases, cation diffusion facilitators (CDF family genes), and RND efflux pumps suggests that T6 employs a combination of metal efflux, periplasmic detoxification, cation sequestration, and mechanisms widely associated with Pb, Zn, and Cd resistance in Gram-negative bacteria [12,58]. Genes related to oxidative stress mitigation, including SOD, catalase, and glutathione-related enzymes, further explain the observed improvement in ROS-inhibiting enzyme activities in rice inoculated with T6 under Pb–Zn stress, demonstrating a coordinated bacterial and rice plant stress alleviation response [14].

In addition, the identification of multiple PGP-associated genes, including those involved in siderophore production, IAA biosynthesis, ACC deaminase activity, and phosphate solubilization, supports the plant growth-promoting effects observed in rice seedlings. These parameters are consistent with the mechanisms by which PGPB enhance nutrient acquisition, root growth, and stress endurance in contaminated soils [59,60]. Taken together, these findings demonstrate that T6 integrates genomic metal resistance determinants with plant-beneficial metabolic pathways, enabling it to survive in Pb–Zn-contaminated environments while simultaneously promoting plant growth. Therefore, T6 represents a promising microbial candidate for microbe-assisted phytoremediation of heavy metal-polluted agroecosystems.

### 4.3. Morphological and Growth Characteristics of the Strain T6

T6 revealed a nearly spherical short rod shape and was Gram-negative. T6 grew best on LB medium, followed by TY and R_2_A media, while it grew slowly in minimal medium. T6 had a very short lag phase. T6 was motile when tested in a semi-solid agar, showing a spreading pattern outward from the stab line. Serratia bacteria can be found in diverse environments like water, soil, on plants and animals as they are opportunist bacteria [61,62,63]. Serratia species were thought to be non-pathogenic environmental bacteria until the discovery of *S. marcescens* being the most common species in the genus, which is an opportunistic pathogen found in many environments [62].

### 4.4. Adoptability of T6 to Different Environments and Resistance to High Concentrations of Heavy Metals

The optimal growth temperature for strain T6 was 28 °C with a wide growth range of between 18 and 37 °C. The vast majority of bacteria are able to live between pH 5.0 and 9.0, while T6 grew well in a range of pH 4.08–9.51. Previously a study reported that the strain *S. rubidaea ED1* was able to grow in a pH range from 4 to 9 [64]. To some extent, differing pH conditions reflect the microorganism’s adaptability to the environment. We further tested T6 for salt tolerance, revealing a high tolerance for strain T6 of up to 9% (*w*/*v*) in R_2_A medium. Another Serratia strain, *Serratia rubidaea ED1*, tolerated up to 8.76% (*w*/*v*) NaCl [64]. For decades, induced salt tolerance in plants has been attributed to the presence of various PGPR strains. T6 is an efficient PGPB able to induce salt tolerance in plants under saline conditions. T6 falls under the moderately halophilic category which grows well between 5 and 10% NaCl, which is very important for developing bio-inoculants that are able to increase crop yields in saline or marginal soils where conventional crops struggle for survival.

Furthermore, we showed that strain T6 was able to grow under both anaerobic and aerobic conditions, indicating that strain T6 is a facultative anaerobe. Based on their oxygen demand and tolerance, microorganisms can be classified into five categories: aerotolerant anaerobes, facultative anaerobes, obligate anaerobes, microaerophiles, and aerobes [65].

T6 grew well under higher concentrations of Zn, Pb, As, Sb, and Cd. Earlier *S. rubidaea ED1,* an endophytic bacterium isolated from *Chenopodium quinoa* roots, displayed resistance to high concentrations of Cu and Cd [64], but T6 was observed to have high resistance to Cu and Cd compared to *S. rubidaea ED1*. Furthermore, *Serratia marcescens BM1*, a potential PGPB strain, was reported to tolerate 300 μM CdCl_2_ [51]. *Serratia marcescens WZ14* was reported to tolerate Pb and Cd in high concentrations [52]. *Serratia marcescens* was reported to have lead, Cd, and chromium metal biosorption [66].

The T6 exhibited tolerance to a variety of heavy metals and metalloids, as evident from its high MIC for Zn, Cd, Pb, As, Sb, etc. The results show that strain T6 had a broad-spectrum metal resistance system, which might involve efflux pumps, metal sequestration, and anti-oxidative defense mechanisms. Previous studies have reported that Serratia species exhibit strong resistance to Zn, Cd, and Pb through bioaccumulation, biosorption, and enzymatic detoxification [67,68]. Relatively high tolerance of T6 to Sb(Ⅲ) and As(Ⅲ) suggests the possible involvement of metalloid efflux systems such as ArsC or ArsB and Sb(Ⅲ) specific transporters [69]. Such multi-metal resistance makes T6 a promising candidate for bioremediation of heavy metal-contaminated soils and waste waters, as well as a phyto-remediating bacterial strain.

### 4.5. Physio-Biochemical Characteristics of the T6

T6 contains genes encoding and producing catalases and esterases but not amylases. T6 produces protease and is able to degrade gelatin. T6 reduces nitrate to nitrite, but it does not produce H_2_S and does not perform mixed-acid fermentation. The gelatin liquefaction test is used to determine the ability of bacteria to produce protease. If bacteria produce protease, they hydrolyze the gelatin in the medium, causing it to lose its gelling ability. T6 was able to liquefy gelatin (protease activity) and hydrolyze esters (Tween hydrolysis), suggesting that T6 produces extracellular enzymes that might be useful for the degradation of proteinaceous and lipid/ester substrates. Such enzymes are often harnessed in waste treatment, bioremediation, and industrial applications (e.g., detergents, leather processing). For instance, protease-producing bacteria have been found to be useful in degrading workplace wastes rich in protein content [70]. The catalase activity of T6 indicates that it is able to deal with reactive oxygen species, which may help T6 in surviving fluctuating oxygen or oxidative stress conditions (e.g., surface environments and processes such as aerobic wastewater treatment and soil). The nitrate reduction capability of T6 indicates that it plays a role in nitrogen cycling, particularly under oxygen-deficient or anaerobic micro-environments. The ability to reduce nitrate allows bacteria to use it as an alternative electron acceptor. This might be vital in soil health by managing nitrogen losses [71]. The broad spectrum of carbon and nitrogen source utilization exhibits metabolic versatility of T6 and also indicates T6 is able to survive in variable conditions where nutrients fluctuate, or in mixed substrate waste streams, making it a promising candidate for applications requiring robust microbial agents.

### 4.6. Plant Growth-Promoting Characteristics of T6

T6 exhibited the strongest IAA production capacity compared to the control and to other strains (T1–T14). T6 was also positive for organic and inorganic phosphorus solubilization as well as having the ability to solubilize potassium and produce siderophores, but T6 was not positive for ACCD activity. Earlier *Serratia marcescens BM1*, a potential PGPB strain, which was isolated from maize rhizospheric soil, was reported to be phosphate-solubilizing and produce IAA [51]. Serratia marcescens WZ14 was reported as a Pb and Cd-tolerant strain possessing plant growth-promoting properties that was isolated from rhizospheric soil [52]. *S. rubidaea ED1*, a halotolerant endophytic bacterium with PGP properties, was isolated from root samples that were collected from *Chenopodium quinoa* [64]. A few other strains of Serratia spp. had been reported to have a positive role in plant growth promotion such as eggplant (*Solanum melongena* L.) [72], pepper (*Capsicum annuum* L.) [73], and cucumber (*Cucumis sativus* L.) [74], under water stress and conditions of high salinity. The beneficial effects of Serratia inoculation have been associated with the activation of various processes in plant cells, such as phytohormone production, zinc solubilization, induction of the plant antioxidant system, as well as expression of beneficial genes [75]. The genome of *Serratia marcescens* RSC-14 possesses efficient PGP properties that alleviated Cd toxicity in host crops [76]. Bacteria producing ACC deaminase activity are known to improve the growth of a variety of plants under stressed conditions of salinity, drought, and heavy metals [43,77]. In our study, 75% isolates were able to produce ACCD.

Evaluating plant-growth-promoting (PGP) traits exclusively under in vitro conditions represents a limitation of the current study. PGP traits of T6 such as IAA production, phosphate/potassium solubilization, and siderophore secretion were assessed under controlled laboratory conditions, which may not fully correspond to their performance in natural soil environments. We also stated that metal tolerance and antioxidative effects were tested in culture media, and their effectuality under field conditions may be different due to interactions with soil microbes, organic matter, and different environmental stimuli. The *pqqABCDEF* operon and PQQ-dependent glucose dehydrogenase presence in T6 suggests its well-characterized gluconic acid-mediated mechanism for inorganic phosphate solubilization; this pathway has been widely reported among efficient P-solubilizing bacteria [78,79]. In addition, the presence of *trpEGDCBA*, *ipdC*, *dhaS*, and *iaaH* indicates that T6 possesses multiple routes for indole-3-acetic acid biosynthesis, in agreement with established IAA-producing pathways in other plant growth-promoting Serratia species [77,80,81,82]. Together, the coexistence of genes encoding functions involved in P-solubilization and IAA-synthesis supports the strong plant growth-promoting potential of strain T6 at the genomic level.

### 4.7. Effects of Strain T6 on Chlorophyll Fluorescence Parameters of Rice Leaves Under Lead and Zinc Stress

Chlorophyll fluorescence parameters are commonly used to monitor the ability of the plant photosynthetic systems to adapt to environmental stress. The main parameters include the maximum photochemical efficiency (Fv/Fm) and the potential activity of PSII (Fv/Fo); the lower these values are, the more severe the photoinhibition. A large number of studies have shown that heavy metal pollution reduces the Fv/Fm and Fv/Fo values of plant PSII [39,80]. Chlorophyll fluorescence parameters Fv/Fm reflect the maximum photochemical efficiency of PSII reaction centers, while Fv/Fo represents the potential activity of PSII. These indicators have been used to evaluate the degree of stress experienced by leaves under adverse environmental conditions [83]. The application of the T6 inoculation increased both Fv/Fm and Fv/Fo ratios in rice leaves under medium to high Pb concentrations. These results indicate that lead stress was able to enhance the Fv/Fm and Fv/Fo ratios in rice, and that the application of strain T6 further improves these parameters under moderate and high Pb stress conditions.

### 4.8. Growth Parameters of Rice Seedlings Affected Under Zinc and Pb Stress

Previous studies have shown that heavy metals display growth promotion at low concentrations and inhibition at high concentrations in several crops [84,85]. In this study, the fresh weight, dry weight, plant height, and root length of rice followed a trend of first increasing and then decreasing with the rising concentrations of Pb and Zn. Here, single Pb or Zn exposure at low concentration had the strongest promoting effect on rice seedling growth. The experimental results demonstrated that strain T6 effectively alleviated Pb stress at high concentrations, thereby protecting and promoting the growth of rice. Under the 150Pb treatment, the application of T6 significantly increased the fresh weight, dry weight, and root length of rice compared with the uninoculated control (*p* < 0.05). However, under 100Pb treatment, this growth-promoting effect was not significant (*p* > 0.05). This may be because, under high concentrations of Pb stress, uninoculated rice plants generate significant amounts of stress-induced ethylene as a physiological stress response, inhibiting growth. Additionally, strain T6 is able to produce indole-3-acetic acid (IAA). Previous studies have shown that IAA can promote root elongation, and this study found that inoculation with strain T6 significantly increased root length compared with the non-inoculated control.

### 4.9. Effect of T6 on Antioxidant Enzymes and Pb, Zn Uptake in Rice Seedlings

MDA, SOD, CAT, and POD are antioxidant enzymes that protect plant cells from damage caused by reactive oxygen species (ROS), generated during stress (like drought, salinity, heavy metals, etc.). Together, SOD, POD/CAT form a defense chain that keeps ROS under control and protects plant cells from oxidative stress. Malondialdehyde (MDA) is the final product of lipid peroxidation in plant cell membranes, able to severely damage the biological membranes of cells. Lipid peroxidation serves as a biochemical marker of oxidative stress in plants; therefore, determining the MDA content in plants can indirectly reflect the degree of membrane damage and the plant’s stress resistance [86]. In our experiment the MDA content in rice roots showed an increasing trend with a rise in exposure to heavy metal(loid)s compared with the control (CK). After inoculation with T6, the MDA content decreased, indicating that T6 altered the MDA accumulation dynamics, thereby alleviating the toxic effects of heavy metal ions on plants.

Earlier studies reported the alleviating effects of DSE (Dark Septate Endophytes) on rice under Pb stress and found that inoculation with *P. fortinii* and *P. fuckelii* significantly reduced MDA content in rice leaves and maintained it at a low and stable level [87]. Similarly, in this study, inoculation with strain T6 under Pb and Zn stress led to a reduction in MDA content, further confirming that T6 mitigates the toxic effects of heavy metal ions on plants by regulating MDA levels. Rice seedlings inoculated with strain T6 (+) significantly enhanced SOD activity compared with non-inoculated ck, particularly under moderate Pb (50 mg·L^−1^) and Zn (100 mg·L^−1^) stress. Some recent studies have revealed the beneficial effects of Serratia spp. strains under saline conditions on various crops such as wheat (*Triticum aestivum* L.) [88], maize (*Zea mays* L.) [89], and quinoa (*Chenopodium quinoa* Willd.) [64].

The PGP activity of *S. marcescens* M8 was observed in wheat at different salinity levels [88]. Moreover, increases in enzymatic and non-enzymatic antioxidant activities (CAT, POD, SOD) and significant reductions in Na^+^ levels and oxidative stress biomarkers (H_2_O_2_ and O_2−_) alleviated the deleterious effects of salinity stress and enhanced wheat growth. We also observed that with the application of T6, the Pb and Zn accumulation slightly decreased in rice root and shoot. Few studies have shown that Serratia species are able to confer both metal and metalloid resistance. *Serratia marcescens* was reported to be able to perform Pb, Cd, and chromium biosorption [66]. This indicates that T6 can be a potential phyto-remediating bacterial strain. This suggests that T6 plays a positive role in stimulating the plant’s enzymatic antioxidant defense system, possibly by reducing oxidative stress through mechanisms such as phytohormone production or improved nutrient uptake.

## 5. Conclusions

The results of this study provided valuable insights into the genomics and physiochemical characteristics underlying the resistance to heavy metals and high salinity of *Serratia ureilytica* T6 and identified potential targets for improving the salt and heavy metal tolerance of *S. ureilytica* T6. *S. ureilytica* T6. Was able to promote plant growth as validated in rice here. The findings of this study may have important implications for the development of new strategies for enhancing the salt and metal(loid) tolerance of microorganisms, which could have significant applications in various fields, including biotechnology and environmental remediation, agricultural land restoration, and the adaptability of crops to survive in saline and heavy metal(loid)-contaminated soils. However, further experimental validation is needed to functionally identify genes and pathways responsible for the adaptation of *S. ureilytica* T6.

Practically, the T6 strain has significant potential for use as a bioinoculant in Zn-Pb-contaminated agricultural soils, where it may improve plant growth, reduce metal-induced oxidative stress, and support safer crop production. In addition, the strain could be applied in microbial-assisted phytoremediation programs, either alone or in combination with metal-accumulating plants, to enhance metal stabilization or uptake. Collectively, our results provide a promising microbial resource for developing sustainable strategies to manage heavy metal pollution in mining-impacted farmlands.

## Figures and Tables

**Table 1:** Initial screening and isolation of strains and determination of the 16S rRNA sequence.

No.	Strains Identified with Highest Similarity	Query Cover	% Identity
T1	*Burkholderia diffusa* R15930	98%	99.79%
T2	*Bacillus cereus* ATCC 14579	97%	99.79%
T3	*Burkholderia pyrrocinia* DSM 10685	97%	99.14%
T4	*Burkholderia anthina* R4183	96%	99.71%
T5	*Burkholderia* cepacia ATCC 24516	97%	99.71%
T6	*Serratia ureilytica FDAARGOS_1089*	97%	99.57%
T7	*Burkholderia territorii* LMG 28158	98%	99.64%
T8	*Bacillus cereus* ATCC 14579	96%	99.94%
T9	*Chryseobacterium gleum* ATCC 35910	97%	99.42%
T10	*Burkholderia pyrrocinia* DSM 10685	96%	99.21%
T11	*LysiniBacillus xylanilyticus* DSM 23493	96%	99.43%
T12	*Chryseobacterium gleum* ATCC 35910	96%	99.57%
T13	*Burkholderia anthina* R4183	97%	99.64%
T14	*Burkholderia territorii* LMG 28158	98%	99.79%

## Data Availability

The original contributions presented in this study are included in the article and Supplementary Materials. Further inquiries can be directed to the corresponding author. The *Serratia ureilytica T6* was submitted to NCBI under Biosample ID (SAMN18104335), Bioproject ID (PRJNA224116), and single complete chromosome (CP071320.1) https://www.ncbi.nlm.nih.gov/nuccore/CP071320.1.

## Supplementary Materials

The following supporting information can be downloaded at: https://www.mdpi.com/article/10.3390/microorganisms13122857/s1, Supplementary Table S1: Stains used in phylogenomic analysis of *Serratia ureilytica* T6. Accessed on July 2025. Supplementary Table S2: Different media used in experiment. Text S1. Detailed genome sequencing of strain T6. Supplementary Table S3: Project information of strain T6. Supplementary Table S4: Putative genes encoding functions involved in heavy metal resistance in strain T6. Supplementary Table S5: Putative genes involved in heavy metals of strain T6. Supplementary Table S6: The ANI (%) values of the strains shown in Figure 1B in the article.

## Abbreviations

The following abbreviations are used in this manuscript:

PGPB	Plant growth-promoting bacteria
ANI	average nucleotide identity
ROS	Reactive oxygen species
MDA	Malondialdehyde
POD	Peroxidase
SOD	Superoxide Dismutase
CAT	Catalase
IAA	indole-3-acetic acid
