# Supplementary material for: Isolation and Genome Analysis of Serratia ureilytica T6, a Heavy Metal(loid)-Resistant and Plant Growth-Promoting Bacterium, from Rice Soil
Source: Microorganisms. 2025 Dec 16;13(12):2857. doi: 10.3390/microorganisms13122857 (PMC12736016; doi:10.3390/microorganisms13122857)
Supplement: Supplementary file 1 [file microorganisms-13-02857-s001.zip › microorganisms-4000761-supplementary.pdf]

Supplementary Table S1. Stains used in phylogenomic analysis of Serratia *ureilytica* T6. Accessed on July 2025.

**Strain Scientific Name**	**Query Cover**	**Percent identity**	**Accession**
Serratia sp. (enterobacteria)	93%	100	CP185812.1
Serratia ureilytica	93%	100	CP196940.1
Serratia ureilytica	93%	100	CP196517.1
Serratia nematodophila DSM21420	93%	100	JPUX0100001
Serratia ureilytica	93%	100	CP196503.1
Serratia marcescens	93%	100	CP047679.1
Serratia ureilytica	93%	100	CP184286.1
Serratia marcescens	93%	100	CP041132.1
Serratia marcescens	93%	100	CP041129.1
Serratia ureilytica	93%	100	CP061078.1
Serratia ureilytica	93%	100	CP061077.1
Serratia sp. JKS000199	93%	100	LT907843.1
Serratia marcescens	93%	100	AP028545.1
Serratia marcescens	93%	100	AP028519.1
Serratia ficaria NCTC12148	93%	100	LT906479.1
Serratia ureilytica	93%	100	CP117886.1
Serratia marcescens	93%	100	OX291765.1
Serratia marcescens	93%	100	OW967878.1
Serratia sp. SCBI	93%	100	CP003424.1
Serratia marcescens	93%	100	AP024916.1
Serratia ureilytica	93%	100	CP071320.1
Serratia ureilytica	93%	100	CP068214.1
Serratia marcescens	93%	99.81	CP197183.1
Serratia marcescens	93%	99.81	CP163407.1
Serratia marcescens	93%	99.81	AP028594.1
Serratia marcescens	93%	99.81	AP028505.1
Serratia marcescens	93%	99.81	AP028480.1
Serratia ureilytica	93%	99.81	CP090028.1
Serratia sp. 2C06	93%	99.61	CP188299.1
Serratia sp. BNK-10	93%	99.61	CP179894.1
Serratia marcescens	93%	99.61	CP028947.1
Serratia marcescens	93%	99.61	CP018926.1
Serratia marcescens	93%	99.61	AP028517.1
Serratia marcescens	93%	99.8	AP028476.1
Serratia ureilytica	93%	99.61	CP074168.1

Serratia ureilytica	93%	99.61	CP060483.1
Serratia ureilytica	93%	99.42	CP084418.1
Serratia marcescens	93%	99.42	CP168445.1
Serratia marcescens	93%	99.42	CP018924.1
Serratia flymuthica	93%	99.42	NC021659.1
Serratia marcescens	93%	99.42	AP028599.1
Serratia marcescens	93%	99.42	AP028586.1
Serratia marcescens	93%	99.61	AP028584.1
Serratia marcescens	93%	99.42	AP028520.1
Serratia marcescens	93%	99.42	AP028499.1
Serratia sp. B1	93%	99.42	CP123616.1
Serratia ureilytica	93%	99.42	CP098030.1
Serratia ureilytica	93%	99.42	CP070508.1
Serratia ureilytica	93%	99.42	CP076651.1
Serratia ureilytica	93%	99.42	CP060276.1
Serratia sp. LS-1	93%	99.22	CP033504.1
Serratia odoripera	93%	99.22	LR134117.1
Serratia marcescens	93%	99.22	OY970407.1
Serratia rubidea	93%	99.22	CP065640.1
Serratia marcescens	93%	99.22	AP028472.1
Serratia ureilytica	93%	99.22	CP091121.1
Serratia marcescens	93%	98.83	CP192733.1
Serratia marcescens	93%	98.83	AP028502.1
Serratia marcescens	93%	99.41	CP025698.1
Serratia sp. 2C06	93%	98.45	CP188298.1
Serratia nevei	93%	97.86	CP186928.1
Serratia sp. HRI	93%	97.86	CP083690.1
Serratia marcescens	93%	98.6	CP053927.1
Serratia sp. BNK-26-b	93%	97.67	CP179903.1
Serratia sp. BNK-23	93%	97.67	CP179900.1
Serratia sp. BNK-26-a	93%	97.67	CP179902.1
Serratia sp. BNK-11	93%	97.67	CP179895.1
Serratia nevei	93%	97.67	CP115015.1
Serratia nevei	93%	97.67	CP115009.1
Serratia marcescens	92%	98.2	CP041134.1
Serratia marcescens	92%	98.2	CP041130.1
Serratia marcescens	92%	98.2	CP041125.1
Serratia marcescens	92%	98.2	CP041123.1

Serratia marcescens	92%	98.2	CP171400.1
Serratia sarumanii	92%	98.2	CP142092.1
Serratia marcescens	92%	98.2	CP018917.1
Serratia marcescens	92%	98.2	CP132197.1
Serratia marcescens	92%	98.2	AP028548.1
Serratia marcescens	92%	98.2	AP028544.1
Serratia marcescens	92%	98.2	AP028529.1
Serratia marcescens	92%	98.2	AP028466.1
Serratia sp. K-E0102	92%	98.2	CP124754.1
Serratia marcescens	92%	98.2	OW849111.1
Serratia marcescens	92%	98.2	CP063238.1
Serratia marcescens	93%	98.2	CP060487.1
Serratia marcescens	93%	97.28	CP166036.1
Serratia nematodiphila	93%	97.28	CP038662.1
Serratia marcescens	93%	97.28	CP143341.1
Serratia marcescens	93%	97.28	CP016032.1
Serratia marcescens	93%	97.28	AP028596.1
Serratia marcescens	93%	97.28	AP028579.1
Serratia marcescens	93%	97.28	CP104092.1
Serratia nematodiphila	93%	97.28	CP100765.1
Serratia marcescens WW4	93%	97.28	CP003959.1
Serratia marcescens	93%	97.28	CP054277.1
Serratia marcescens	92%	98	CP033623.1
Serratia marcescens	92%	98	CP029715.1
Serratia marcescens	92%	98	CP029746.1
Serratia marcescens	92%	98	CP150016.1
Serratia marcescens	92%	98	AP028533.1
Serratia marcescens	92%	98	AP024847.1
Serratia microhaemolytica	92%	98	SBEF01000001
Serratia fonticola	92%	98	AVAH100001
Pseudomonas profundi M5	92%	96.2	VTPZ0100001

Supplementary Table S2. Different media used in experiment.

Medium	Composition (per liter)	Purpose
Nutrient Agar (NA)	Peptone 5 g; Beef extract 3 g; NaCl 5 g; Agar 15 g	General-purpose bacterial growth
LB Broth / LB Agar	Tryptone 10 g; Yeast extract 5 g; NaCl 10 g; Agar 15 g (for solid), pH 7.0	Routine cultivation, cloning
R2A Medium	Yeast extract 0.5 g; Proteose peptone 0.5 g; Casamino acids 0.5 g; Glucose 0.5 g; Starch 0.5 g; Sodium pyruvate 0.3 g; K_2HPO_4 0.3 g; $MgSO_4 \cdot 7H_2O$ 0.05 g; Agar 15 g (solid), pH 7.2	Isolation of slow-growing environmental bacteria
Minimal Salt Medium (MSM)	KH_2PO_4 1.5 g; Na_2HPO_4 3.5 g; NH_4Cl 1 g; $MgSO_4 \cdot 7H_2O$ 0.2 g; $CaCl_2$ 0.01 g; Glucose 2 g, pH 7.0	Metal tolerance studies, selective growth

Detailed Genome Sequencing of Strain T6

For genome sequencing, the total genomic DNA was extracted using the TIANamp bacteria DNA isolation kit as described by the manufacturer (TianGen Biotech, Beijing Co., Ltd.). After evaluating the quality and amount of the extracted DNA, it was processed for Oxford Nanopore, including sample quality detection and library construction, followed by integrity and quality checks using Nanodrop, Qubit, and 0.35% agarose gel electrophoresis. Large DNA fragments were recovered using the BluePippin fully automatic nucleic acid recovery system. The library was generated using a ligation kit (SQK-LSK109) and forwarded for sequencing. Guppy software (version 0.17.1) of Oxford Nanopore Technologies was employed for the base calling. The Nanofilt tool (version 2.8.0) was used to filter the reads. Any readings with a mean Q-score below 10 were eliminated from subsequent analysis. The filtered reads were assembled using Canu v1.5(2). The assembly results were corrected using the third-generation reads using Racon v3.4.3 software. Circularization and start site adjustment were performed using Circlator v1.5.5 software. Gene prediction was performed using the software Prodigal v2.6.3. Three types of rRNAs in the genome were predicted based on the covariance models using Infernal v1.1.3, while tRNAs were predicted using tRNAscan-SE v2.0. CRT v1.2 software was used to perform CRISPR prediction on the genome. The functional annotation mainly included general databases such as Nr, Uniprot, COG, KEGG, and other databases, as well as proprietary database annotations such as CAZyme, PHI, and CARD. Genome map analysis included a circular genome map generated using the genome map display tool.

Genome Assembly

Filtered subreads were assembled using Canu v1.5 software. The assembly results were corrected using third-generation subreads with Racon v3.4.3. Circularization and starting site adjustment were performed with Circlator v1.5.5. Further error correction was carried

out using second-generation data with Pilon v1.22, resulting in a more accurate genome for subsequent analysis.

Basic Information on Genome Sequencing of Strain T6

The basic sequencing information of strain T6 is shown in Table 3, which includes details on sequencing quality, library construction, assembly methods, and other essential data related to the genome of strain T6. The complete genome sequence of strain T6 has been submitted to the GenBank database, with the accession number, CP071320.

Supplementary Table S3. Project information of strain T6.

MIGS ID	Property	Term
MIGS 31	Finishing quality	High-Quality Draft
MIGS 29	Sequencing platforms	Nanopore
MIGS 31.2	Fold coverage Illumina	357X
MIGS 30	Assemblers	Canu
MIGS 32	Gene calling method	Glimmer3
	Locus Tag	J0X03
	N50 length	21950
	N90 length	4225
	Non-coding rRNA	34
	Non-coding tRNA	26
	GenBank ID	CP071320
	GenBank Date of Release	2021.03
	BioProject	PRJNA659846
	Project relevance	Bioremediation

Analysis of Genome Annotation Results for Strain T6

By comparing genes against databases for annotation, the functions and related descriptive information of genes were identified, providing an overall functional classification of the gene set. This facilitates the identification of target functional genes for subsequent studies. We used three databases, GO, COG, for annotation. The statistical summary of the gene annotation results for strain T6 is presented below.

Supplementary Table S4. Gene set annotation result statistics of strain T6.

Sample	Functional Database	Number of genes	100< = sequence	Sequence length >= 300

			length<300	
	eggNOG_Annotation	4,381	2,029	2,115
	GO_Annotation	3,604	1,645	1,794
	nr_Annotation	4,633	2,137	2,148
T6	Pfam_Annotation	4,303	1,975	2,092
	Swissprot_Annotation	3,554	1,514	1,872
	TrEMBL_Annotation	3,554	1,514	1,872
	All_Annotated	4,633	2,137	2,148

Supplementary Table S5. Putative genes involved in heavy metals of strain T6.

Category	Putative function	Number	Feature_id
Lead resistance	Lead, cadmium, zinc, and mercury transporting ATPase	3	Fig|300181.100.peg.3620<br>Fig|300181.100.peg.3667<br>Fig|300181.100.peg.4499
Zinc resistance	ZhuABC	6	Fig.|300181.100.peg.1985<br>Fig.|300181.100.peg.3657<br>Fig.|300181.100.peg.1984<br>Fig.|300181.100.peg.3656<br>Fig.|300181.100.peg.1986<br>Fig.|300181.100.peg.3655
	Zinc uptake regulation protein, Zur	1	Fig.|300181.100.peg.382
	Zinc transporter, ZitB	1	Fig.|300181.100.peg.3178
	Transcriptional regulator, MerR family	1	Fig.|300181.100.peg.3168
Cobalt-zinc-cadmium resistance	Cobalt-zinc-cadmium resistance protein Czc	3	Fig.|300181.100.peg.64<br>Fig.|300181.100.peg.2255<br>Fig.|300181.100.peg.3450
	DNA-binding heavy metal response regulator	1	Fig.|300181.100.peg.3222

Supplementary Table S6. The ANI (%) values of the strains shown in Fig. 1b in the article.

Strain (row/col)	1	2	3	4	5	6	7	8	9	10	11	12	13	14
**1 S. microhaemolytica ZS-11**	**100**	72	73	73	74	75	75	75	76	74	76	75	76	75
**2 S. ureilytica T6**	72	**100**	98	95	92	79	80	79	81	80	81	80	79	78
**3 S. ureilytica FDAARGOS_1089**	73	98	**100**	96	93	80	81	80	82	81	82	81	80	79
**4 S. nematodiphila DSM 21420**	73	95	96	**100**	94	81	82	81	83	82	83	82	80	80
**5 S. marcescens ATCC 13880**	74	92	93	94	**100**	82	82	82	84	83	84	83	82	81
**6 S. odorifera NCTC11214**	75	79	80	81	82	**100**	94	80	79	79	80	80	79	78
**7 S. odorifera FDAARGOS_353**	75	80	81	82	82	94	**100**	81	80	81	81	80	79	79
**8 S. grimesii NBRC 13537**	75	79	80	81	82	80	81	**100**	86	85	86	85	83	82

**9 S. proteamaculans 336X**	76	81	82	83	84	79	80	86	**100**	95	92	90	85	83
**10 S. quinivorans NCTC13188**	74	80	81	82	83	79	81	85	95	**100**	90	89	84	82
**11 S. ficaria NCTC12148**	76	81	82	83	84	80	81	86	92	90	**100**	95	86	83
**12 S. plymuthica S13**	75	80	81	82	83	80	80	85	90	89	95	**100**	87	84
**13 S. rubidaea FDAARGOS_926**	76	79	80	80	82	79	79	83	85	84	86	87	**100**	92
**14 S. fonticola LMG 7882**	75	78	79	80	81	78	79	82	83	82	83	84	92	**100**
